# Direct Time-Domain Eduction of Acoustic Liner Impedance in Turbulent Grazing Flows

**DOI:** 10.1017/jfm.2026.11969

**Published:** 2026-09-04

**Authors:** Rémi Roncen, Ludovic Ambrosiani, Angelo Paduano, Francesco Avallone, Fabien Méry, Estelle Piot

**Affiliations:** 1 DMPE, ONERA, Université de Toulouse, 31000, Toulouse, France; 2 Department of Mechanical and Aerospace Engineering, Polytechnic of Turin, Turin, Italy

**Keywords:** Aeroacoustics, Wave-turbulence interactions, Channel flow, Noise Control

## Abstract

Acoustic liners are passive sound-absorbing materials widely used in engineering applications and are commonly characterised by their acoustic impedance. In the presence of high sound pressure levels or grazing flows, the impedance is modified by local nonlinear effects. Conventional impedance measurements are typically obtained through frequency-domain eduction techniques, which infer impedance from its effect on measurable acoustic quantities and therefore rely on assumptions regarding wave propagation and near-wall acoustic-flow interactions. In this paper, a time-domain impedance eduction approach based on instantaneous wall-normal velocity measurements is developed to investigate the nonlinear response of acoustic liners under grazing-flow conditions. Unlike conventional methods, the proposed approach does not rely on a wave-propagation model and reconstructs the liner impedance directly from the local velocity field. The method is assessed using both laser Doppler velocimetry measurements and high-fidelity numerical simulations. The resulting impedance estimates show good agreement with classical predictions while revealing that a significant part of the flow effect originates from turbulence-induced wall-normal velocity fluctuations acting through the same nonlinear mechanisms as high-amplitude acoustic excitation. Furthermore, the commonly reported upstream–downstream impedance mismatch largely disappears when wave-propagation modelling is removed from the eduction process, suggesting that this discrepancy primarily arises from modelling assumptions rather than from an intrinsic dependence of the liner impedance on the direction of acoustic propagation.

## 1 Introduction

Aeroacoustic liners are widely used passive noise control devices (Ma & Su 2020), typically consisting of perforated face sheets backed by cavities. Their performance is commonly described in the frequency domain by the acoustic impedance (Bauer 1977), a complex-valued quantity that defines a liner’s capacity to dissipate acoustic energy within a system.

Impedance is not a fixed property: it varies significantly with operating conditions, most notably under high sound pressure levels (SPLs) or when exposed to a shear grazing flow. In particular, vortex shedding at liner perforations, either acoustic or turbulent in their origin, can generate locally nonlinear behaviours at SPLs far below those required for global nonlinear wave propagation (Zhang & Bodony 2012; Ingard & Ising 1967; Melling 1973; Tam *et al.* 2001; Jing & Sun 2002). The introduction of SPL-dependent nonlinearities complicates conventional measurement and optimization approaches, as it requires accounting for a spatially varying impedance when the pressure field is non-uniform (Lafont *et al.* 2020; Roncen *et al.* 2022). Fundamentally, the emergence of a nonlinear term is associated with strong acoustic particle acceleration and large velocity gradients at the inlet and outlet of the perforated facesheet—typically of low porosity (porosity ≤ 10%)—as a direct consequence of mass-flow conservation.

The presence of a sheared mean flow can strongly influence the value of the impedance (Guess 1975; Cummings 1986*a*; Kirby & Cummings 1998; Jones *et al.* 2010), making its identification particularly challenging. Even a slight change in the displacement boundary layer thickness can lead to widely different impedances (Quintino *et al.* 2025). It is also worth noting that the presence of flow modifies the optimal impedance of a system designed to maximise sound attenuation (Rice 1969). Fundamentally, the interaction between the flow and the liner impedance remains poorly understood to date. It appears that the mean flow penetrates the perforated plate and alters the way the acoustic wave “sees” the perforations. This phenomenon, commonly referred to as the vena contracta effect, reduces the effective cross-sectional area available for acoustic motion within the liner facesheet and therefore tends to increase the liner resistance, in a manner similar to that observed under steady bias flow (Jing & Sun 1999). This behaviour bears strong resemblance to flow through porous media governed by Darcy**–**Forchheimer regimes, although this analogy is still rarely discussed in the acoustics literature (Shahzad *et al.* 2022, 2023; Hoang *et al.* 2024), despite numerical results showing the difference between laminar and turbulent flow effects on an orifice impedance (Zhang & Bodony 2016a). In addition, the flow convects acoustically-induced vortical structures (at high SPL), which may further alter the acoustic response of downstream perforations. For instance, recent studies by Paduano *et al.* (2026), carried out with a lattice Boltzmann method very large-eddy simulation (LBM-VLES), have evidenced that acoustic-induced velocity during the outflow phase generates a jetting-like mechanism at the orifice mouth, which displaces the near-wall fluid away from the wall and modifies the downstream boundary layer resulting in a different acoustic-induced velocity at the downstream cavities, which in turn might affect the dissipation mechanism (Howe 1996; Scarano *et al.* 2025).

These external dependencies–high SPL and shear grazing flow– motivate continued efforts to reliably determine impedance in realistic flow and acoustic environments.

Direct measurement of impedance is generally not possible in the presence of flow, except with the intrusive and somewhat tricky in-situ method (Dean 1974; Bonomo *et al.* 2024). Instead, researchers rely on indirect approaches known as impedance eduction. These methods combine experimental measurements—typically acoustic pressures or velocity fields in flow ducts—with wave propagation models, and determine the impedance that best reproduces the observed data (Watson *et al.* 1999; Jones *et al.* 2003; Jing *et al.* 2008; Primus *et al.* 2013; Spillere *et al.* 2017; Troian *et al.* 2017; Weng *et al.* 2018; Howerton *et al.* 2019; Qiu *et al.* 2024). Most eduction techniques are rooted in the frequency-domain, solving harmonic propagation problems given mean flow properties and an impedance boundary condition. Optimization algorithms (Primus *et al.* 2013; Watson & Jones 2013) or Bayesian inference (Roncen *et al.* 2019) strategies are then employed to identify the impedance that either minimises the discrepancy or, equivalently, maximises the likelihood between experimental observations and model predictions. In the Bayesian framework, this procedure explicitly accounts for uncertainties in the model and measurements.

The pioneering observations of Renou & Aurégan (2011) revealed that, in the presence of flow, impedance inferred from upstream versus downstream wave propagation was not identical, challenging the long-held assumption that impedance is an intrinsic material property. This triggered a broad research effort into whether impedance alone suffices to capture the complex physics of liner-flow interactions. In particular, several studies investigated whether the mismatch could originate from limitations of the impedance boundary condition itself, leading to more sophisticated formulations accounting for finite boundary-layer thickness effects (Khamis & Brambley 2016).

A key drawback of eduction strategies is that any deficiency in the propagation model or in the boundary-condition modelling is implicitly absorbed into the retrieved effective impedance. As a result, educed impedance can depend not only on the duct geometry or experimental method but also on the direction of acoustic waves relative to the mean flow, depending on the method used (Roncen *et al.* 2020). However, due to the tight coupling between the wave propagation modelling and the educed impedance, it has so far not been possible to sort out the upstream-downstream mismatch in terms of wave propagation modelling bias and genuine physical effects changing the impedance, such as the momentum transfer of Schulz *et al.* (2017). Weng *et al.* (2018) verified that the mismatch was not caused by neglecting viscosity in the wave propagation solvers used for eduction, while Roncen *et al.* (2022, §3.2) showed that nonlinear effects on the liner can lead to a spatially varying impedance that differs depending on the excitation direction. Nevertheless, the upstream**–**downstream mismatch has also been observed in contexts where nonlinear liner effects can be neglected, indicating that the full picture remains unresolved. Furthermore, Roncen (2025*b*) suggested in a preliminary experiment that turbulence-induced noise may in part trigger the same nonlinearities as high SPL, further blurring the line between flow effects and acoustic response.

Taken together, these observations point towards two unresolved questions: the physical origin of the flow effect on liner impedance and the origin of the upstream–downstream impedance mismatch. The present work introduces a new time-domain methodology specifically designed to investigate these questions, the DIR-TDIBC (dynamic impulse response time-domain impedance boundary condition) framework (Roncen & Cardesa 2023). The DIR-TDIBC is based on instantaneous wall-normal velocity measurements, and, unlike conventional methods, reconstructs the liner impedance directly from the local velocity field without relying on a wave-propagation model. The method is assessed using both laser Doppler velocimetry (LDV) measurements and high-fidelity numerical simulations. Beyond its practical use as an impedance-evaluation tool, the method provides a means of isolating local liner-flow interactions from wave-propagation modelling effects, thereby providing a basis from which to revisit the two open questions identified above.

The paper is organised as follows. The theoretical background elements required for the modelling of the impedance of acoustic liners are introduced in Section 2. The DIR-TDIBC approach is described in Section 3. Experimental and numerical configurations are showcased in Section 4. The method is applied to an LDV experiment conducted in an aeroacoustic bench, in Section 5, and to numerical data obtained via LBM-VLES in Section 6. A discussion on the limits and merits of the method is given in Section 7. Conclusions are drawn in Section 8.

## 2 Theoretical background

This section introduces the theoretical background of liner impedance modelling, with a particular focus on frequency-domain quantities and models, as this constitutes a necessary step before transitioning to a time-domain approach. Section 2.1 presents the classical frequency-domain formulation of a single-degree-of-freedom (SDoF) liner, which serves as the reference liner material throughout this study, owing to its continued widespread use in the liner industry. Readers interested in more complex and efficient liners are referred to Jones *et al.* (2022) for a review. Sections 2.3 and 2.4 then examine the influence of nonlinear and flow effects on impedance, respectively, and provide empirical model descriptions for both. Although only SPL-related nonlinear effects are incorporated into the time-domain approach proposed in this work, an empirical model describing flow effects is retained as a basis for comparison with previous studies.

### 2.1 Acoustic liner impedance

The normalised surface impedance, denoted by *Z* in the frequency domain, is defined as the ratio of acoustic pressure $\tilde{p}$ to the normal acoustic particle velocity ${\tilde{v}}_{n}$ at the liner surface, scaled by the characteristic impedance of the ambient medium, *ρ*_*f*_ *c*_*f*_, where *ρ*_*f*_ is the fluid density and *c*_*f*_ the speed of sound. It is commonly expressed in terms of its real and imaginary parts—resistance $\tilde{r}$ and reactance $\tilde{\chi }$—as

(2.1) $$Z(\omega )=\frac{\tilde{p}(\omega )}{\rho_{f}c_{f}{\tilde{v}}_{n}(\omega )}=\tilde{r}(\omega )+\text{j}\tilde{\chi }(\omega ),$$

where *ω* = 2*π* *f* is the angular frequency in rad/s, with *f* the frequency in Hz and j the imaginary unit.

**Figure 1:**
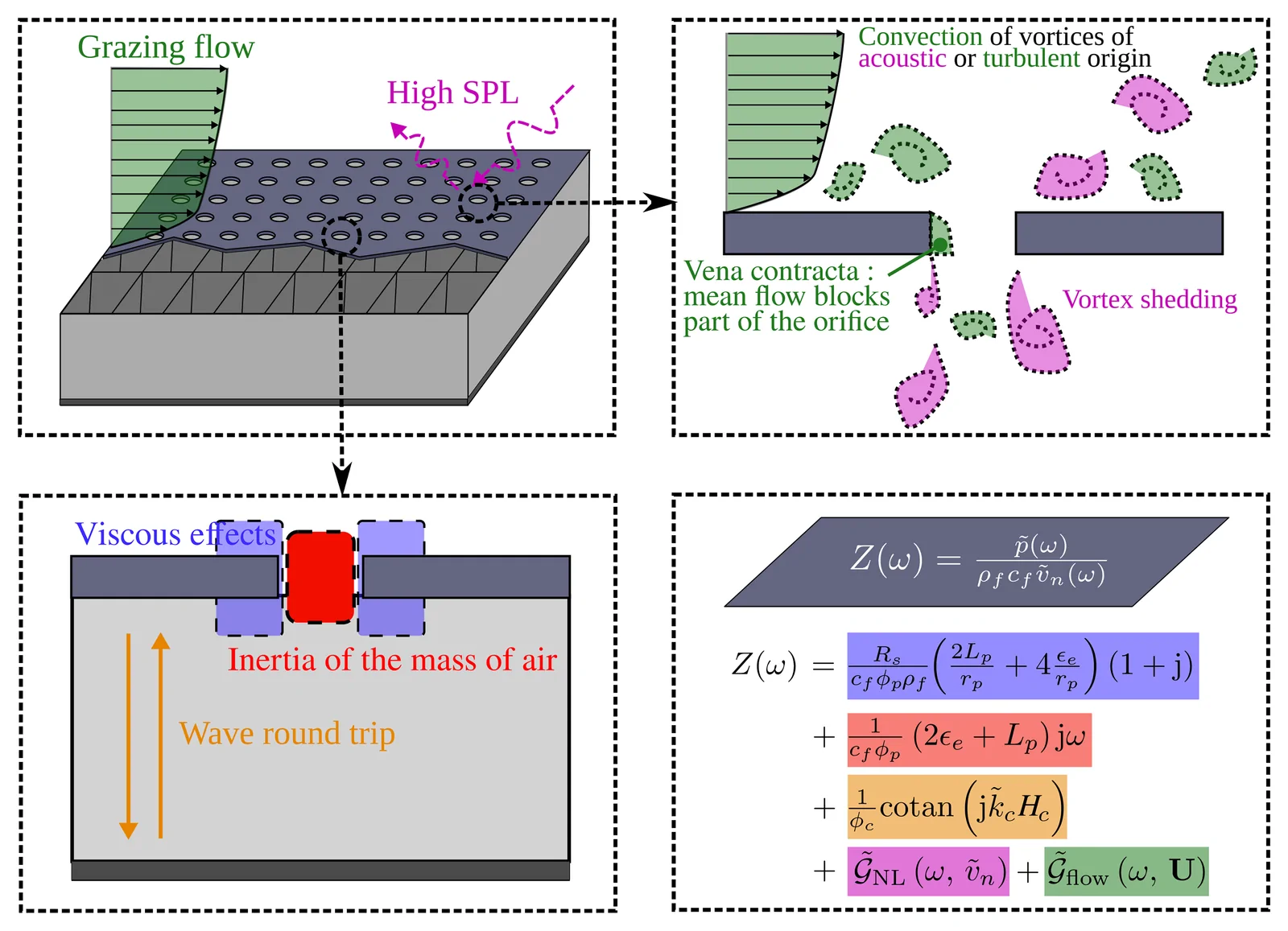
(Colour online) Graphical summary of the physical effects influencing the impedance. a) Representation of an SDoF liner. b) Representation of nonlinear and flow effects. c) Representation of physical effects in play for the definition of the linear impedance. d) A colour code is followed to associate each physics with the terms of the homogenised impedance in Eq. 2.2.

It should be emphasised that $\tilde{p}$ and ${\tilde{v}}_{n}$ are typically interpreted as homogenised quantities, such that the fine-scale velocity variations within the perforations are not explicitly resolved. Instead, the liner surface is treated as acoustically smooth, and the impedance represents an effective boundary condition, that includes all the physical effects pertaining to the sound wave interaction, as shown in the graphical summary in figure 1 for a particular liner, the SDoF, as detailed in the sections below.

### 2.2 Linear impedance of an SDoF liner

Complex liners are often represented in the frequency domain as assemblies of elementary units using transfer matrix methods (Allard & Atalla 2009, Chap. 11). For clarity, however, a more compact SDoF formulation is adopted in this work, where only one perforated plate and one cavity are considered. The normalised impedance of an SDoF liner is expressed following the approach of Atalla & Sgard (2007, Eq. 8), with the cavity wavenumber ${\tilde{k}}_{c}$ determined using the formulation of Bruneau (2006, § 3.7):

(2.2a) $$\begin{array}{l} Z_{0}(\omega )=\frac{R_{s}}{\rho_{f}c_{f}\phi_{p}}\left(\frac{2L_{p}}{r_{p}}+4\frac{\epsilon_{e}}{r_{p}}\right)\frac{(1+\text{j})\sqrt{2}}{2}\\ \;+\frac{\left(2\epsilon_{e}+L_{p}\right)}{c_{f}\phi_{p}}\text{j}\omega +\frac{1}{\phi_{c}}\coth\left(\text{j}{\tilde{k}}_{c}H_{c}\right) \end{array}$$

(2.2b) $$\epsilon_{e}=0.85r_{p}\left(1-1.14\sqrt{\phi_{p}}\right),\;\text{Correction}\;\text{length}\;$$

(2.2c) $$R_{s}=\sqrt{\eta \omega \rho_{f}},\;\text{Surface}\;\text{resistance}\;$$

(2.2d) $$\text{j}{\tilde{k}}_{c}=\frac{\text{j}\omega }{c_{f}}{\left[\frac{1+(\gamma -1)\Phi \left(k_{\kappa }r_{c}\right)}{1-\Phi \left(k_{v}r_{c}\right)}\right]}^{1/2},\;\text{Cavity}\;\text{wavenumber}\;$$

(2.2e) $$\Phi (s)=\frac{2}{s}\frac{I_{1}}{I_{2}}(s).$$

Here, an *e*^+j*ωt*^ convention is assumed, and the formulation is valid for *ω* > 0. The symbols ***L***_*p*_, *ϕ*_*p*_, and *r*_*p*_ denote, respectively, the thickness, porosity, and hole radius of the perforated plate, while *H*_*c*_, *ϕ*_*c*_, and *r*_*c*_ describe the cavity’s height, porosity and hydraulic radius. Here *I*_*n*_ are modified Bessel functions of the first kind of order *n*, with $k_{v}=\sqrt{\text{j}\omega /v}$ and $k_{\kappa }=\sqrt{\text{j}\omega /\kappa }$. The ambient fluid is characterised by the speed of sound *c*_*f*_, density *ρ*_*f*_, heat capacity ratio *γ*, dynamic viscosity *η*, kinematic viscosity *v*, and thermal conductivity *k*.

### 2.3 Nonlinear effects

Although impedance is formally a concept tied to linear systems, in practice it is often extended to describe nonlinear behaviour in the frequency domain when harmonic excitation is considered. While this extension is not strictly rigorous, it has proven effective for characterizing liner performance at high SPL (Billard *et al.* 2020).

Most nonlinear impedance models (Ingard & Ising 1967; Melling 1973; Guess 1975; Cummings 1986b; Temiz *et al.* 2016; Boden *et al.* 2024) adopt an additive formulation in which the total impedance is expressed as

(2.3) $$Z_{\text{tot}}\left(\omega ,{\tilde{v}}_{n}(\omega )\right)=Z_{0}(\omega )+G_{\text{NL}}\left(\omega ,{\tilde{v}}_{n}(\omega )\right),$$

where *Z*_0_ (*ω*) is the baseline linear impedance, and 𝒢_NL_ is a nonlinear contribution that depends on frequency, perforation geometry, plate porosity, and the normal particle velocity ${\tilde{v}}_{n}(\omega )$ at the liner surface. Depending on the author, the particle velocity may be defined in different ways, based either on the root-mean-square (rms) value of the particle velocity over time or on its peak magnitude. In order to apply the time-domain methodology presented in this work, an rms-based formulation must be adopted.

In this study, the nonlinear correction 𝒢_NL_ is split into real and imaginary parts using empirical models recommended by Boden *et al.* (2024). The real component is estimated using the formulation of Temiz *et al.* (2016, Eq. 13), while the imaginary part is modeled with the correction of Shah *et al.* (2024, Eq. 14). For reference, these *empirical* models are provided here for *ω* > 0:

(2.4a) Re(GNL)=2|v˜n|ϕp2cf12Cvc211+2St⋅(1+0.06e3.74Sh),

and

(2+.4b) $$\text{Im}\left(G_{\text{NL}}\right)=\frac{k_{f}r_{p}}{\phi_{p}}\left[-\left(0.6+1.6S_{h}^{-1}+0.015S_{h}\right)\left(1-\frac{1}{1+0.013S_{t}^{-2.5}S_{h}^{2}}\right)\right],$$

where *k*_*f*_ = *ω*/*c*_*f*_ is the air wavenumber, *C*_vc_ = 0.8 is the vena contracta coefficient, *S*_*h*_ is the shear wave number and *S*_*t*_ is the Strouhal number based on the particle velocity rms inside the perforation ${\tilde{u}}_{p}(\omega )=\left|{\tilde{v}}_{n}(\omega )\right|/\phi_{p}$:

(2.5a) $$S_{h}=r_{p}\sqrt{\frac{\omega }{v}},$$

(2.5b) $$S_{t}=\frac{2\omega r_{p}}{\sqrt{2}{\tilde{u}}_{p}(\omega )}.$$

The real part of the proposed nonlinear model is quite sensitive to the choice of the vena contracta coefficient *C*_vc_. In the present work, a value of *C*_vc_ = 0.8 was selected, slightly higher than the typical value reported for sharp-edged orifices (*C*_vc_ ≈ 0.75), in order to account for the bevelling of the perforations in the liner samples considered in the present work (Moers *et al.* 2011, Chap. 4).

### 2.4 Flow effects

Typically, in the presence of a shear grazing flow, an additional contribution to the impedance is added, as

(2.6) $$Z_{\text{tot}\;}\left(\omega ,{\tilde{v}}_{n}(\omega )\right)\leftarrow Z_{\text{tot}\;}\left(\omega ,{\tilde{v}}_{n}(\omega )\right)+G_{\text{flow}\;}\left(U_{0}\right),$$

where 𝒢_flow_ (**U**_**0**_**)** is usually a real function of the liner’s flow-facing porosity and of the flow profile **U**_**0**_ (Guess 1975; Yu *et al.* 2008).

A common simplification is to consider the Goodrich model of Yu *et al.* (2008) for a liner placed in a flow duct, as

(2.7) $$G_{\text{flow}\;}\left(U_{0}\right)=\frac{M_{\text{b}}}{\phi_{p}\left(2+1.256\frac{\delta^{⋆}}{d}\right)}M_{\text{b}},$$

where *M*_b_ is the bulk Mach number and *δ*^★^ is the boundary-layer displacement thickness. Parameter *δ*^★^ is defined as

(2.8) $$\delta^{⋆}=\int_{0}^{H/2}\left(1-\frac{U(y)}{U_{0}}\right)dy,$$

where H is the height of the duct.

In the remainder of this work, impedance evaluation within the time-domain framework *does not* incorporate explicit flow corrections through the Goodrich model or related formulations of Eq. 2.6. Instead, only the linear and nonlinear contributions of Eq. 2.3 are retained. The wall-normal component of the turbulence-induced velocity field is assumed to affect the effective impedance exclusively through the nonlinear mechanisms described by Eq. 2.4.

This approach constitutes a significant departure from classical studies, in which flow effects are typically represented through non-local static quantities such as the Mach number. Here, following the initial exploration of Roncen (2025*b*, Sec. 5), we hypothesise that the empirical correlations proposed in earlier works are, at least in part, directly driven by wall-normal velocity fluctuations induced by turbulence. In this view, these fluctuations modulate the impedance in a manner analogous to acoustic velocity fluctuations.

To test this hypothesis, the wall-normal velocity fields measured experimentally or obtained numerically above a liner—encompassing both hydrodynamic and acoustic perturbations—are injected into a time-domain representation of the nonlinear impedance.

If the hypothesis holds, the resulting impedance should coincide with that predicted by classical empirical models based on Mach number scaling, such as the Goodrich model, which is used here solely for comparison owing to its simplicity and widespread adoption.

### 2.5 The reflection coefficient

The numerical method employed throughout this work is formulated in terms of the liner’s reflection coefficient. The reflection coefficient, sometimes referred to as the scattering operator *R*, is bounded within the complex unit circle, which makes it particularly convenient and numerically stable for simulation purposes (Delorme *et al.* 2005, §3.3; Ventribout 2006, §2.3; Monteghetti 2018, §5.3). Moreover, *R* is bijectively related to the normalised impedance through the relation

(2.9) $$R\left(\omega ,{\tilde{v}}_{n}(\omega )\right)=\frac{Z_{\text{tot}}\left(\omega ,{\tilde{v}}_{n}(\omega )\right)-1}{Z_{\text{tot}}\left(\omega ,{\tilde{v}}_{n}(\omega )\right)+1}.$$

In practice, however, results are reported in terms of impedance, as this representation is the most commonly used and readily interpreted within the liner community.

## 3 The DIR-TDIBC methodology

**Figure 2:**
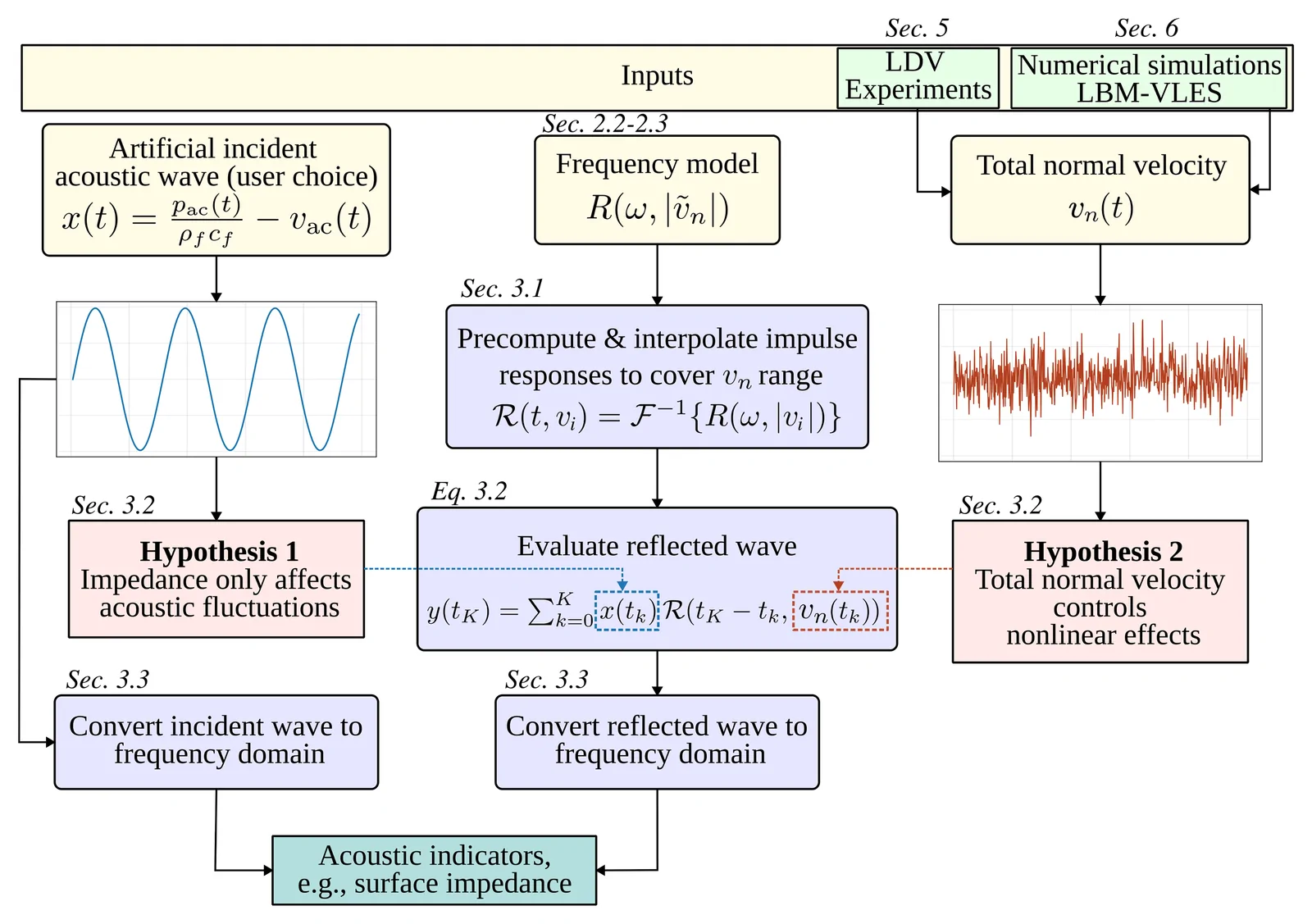
Graphical summary of the DIR-TDIBC method applied in a nonlinear context, where the normal velocity– measured by LDV or via probes in high-fidelity simulation– is used to replay the nonlinear behaviour during the convolution of Eq. 3.2.

This section is dedicated to the new methodology developped in this work, and on how it can be applied to practical cases of interest. Section 3.1 addresses the conversion from frequency to time domain, as done by the DIR-TDIBC method. Section 3.2 presents how the method is applied in a context where hydrodynamic and acoustic perturbations coexist, as well as the associated hypotheses that were made. The post-treatment of time-domain signals into the frequency domain is discussed in Section 3.3. A schematic summarizing the DIR-TDIBC method is shown in figure 2.

### 3.1 The dynamic impulse response approach to TDIBC

In this work, the liner’s impulse response–defined as the inverse Fourier transform of its response to a unit impulse–is used as the fundamental building block. In discrete form, the reflection coefficient can thus be represented as a finite-length impulse response array ℛ, evaluated as

(3.1) $$ℛ\left(t,v_{n}(t)\right)={ℱ}^{-1}\left\{R\left(\omega ,v_{n}(t)\right)\right\},$$

where ℱ^−1^ is the inverse Fourier transform operator, and where *v*_*n*_ is the instantaneous wall-normal velocity above the liner (represented by the red curve in figure 2). Consequently, the impulse response evolves continuously with the instantaneous value of *v*_*n*_, and each spatial location on the liner is associated with its own time-varying impulse response.

This choice leverages the flexibility of the impulse response: any time-domain signal can be represented as a sequence of instantaneous values, so knowing the liner’s response to a unit impulse allows its response to arbitrary inputs to be predicted by summing the contributions of each discrete pulse.

Strictly speaking, the impulse response is not uniquely defined for a nonlinear system. In the present work, it is recalculated at each time step to account for the instantaneous velocity at the perforation, which is assumed sufficient to capture the system’s nonlinearity. Conceptual limitations of this approach were discussed by Roncen (2025*b*, Sec. 3.3.1).

In numerical solvers, impulse responses can be efficiently accumulated using arrays: a reference array stores the precomputed response, which is then sequentially processed into a secondary array to accumulate the boundary condition’s future response. This DIR-TDIBC approach, first proposed by Roncen & Cardesa (2023), extends naturally to nonlinear operators and can handle both harmonic and broadband signals, including those with discontinuities.

The time-local *dynamic* convolution between an incident wave *x* (*t*) and the reflection coefficient is expressed as

(3.2) $$y\left(t_{K}\right)=\sum_{k=0}^{K}x\left(t_{k}\right)ℛ\left(t_{K}-t_{k},v_{n,k}\right),$$

where *x* (*t*) denotes the incident wave amplitude, *y* (*t*) the reflected wave amplitude, *ℛ* the (generally nonlinear) impulse response kernel, and *v*_*n,k*_ the instantaneous wall-normal velocity at time *t*_*k*_.

Using the instantaneous velocity rather than its rms value provides superior temporal resolution for modelling complex, time-varying excitations, as demonstrated by Roncen (2025*b*). In the present framework, the incident amplitude *x*(*t*) is prescribed by the user, typically as a sinusoidal signal at the acoustic excitation frequency (see the blue signal in figure 2).

This differs from the approach of Roncen (2025*b*), in which the incident amplitude was obtained through a wave-sorting procedure and subsequently combined with the reflected wave to evaluate *v*_*n,k*_. Here, the particle velocity governing the nonlinear behaviour of the impulse response is not derived from *x* (*t*) and *y*(*t*) ; instead, it is directly extracted from velocity measurements and imposed as an external input to the time-domain model, as represented by the red dashed arrow connecting the total normal velocity to the convolution evaluation in figure 2.

At each simulation step *t*_*k*_, the impulse response is obtained via the inverse Fourier transform of *R*, using the instantaneous velocity in place of the frequency-domain velocity in Eqs. 2.3 and 2.9:

(3.3) $$ℛ\left(t,v_{n,k}\right)=\frac{1}{2\pi }\int_{0}^{\infty }R\left(\text{j}\omega ,\left|v_{n,k}\right|\right)e^{\text{j}\omega t}\text{d}\omega .$$

Precomputing responses for representative values of the total wall-normal velocity *v*_*i*_ (see figure 2) and interpolating between them avoids recalculation at each time step, enabling efficient and accurate simulations.

In practice, 200 discrete values of *v*_*i*_ were used, uniformly distributed between the minimum and maximum of |*v*_*n*_| observed in the signal. For each value, a corresponding impulse response was precomputed, forming a lookup table used during the time-domain convolution. At each time step, the appropriate response is obtained via linear interpolation in *v*_*i*_. The accuracy of this interpolation strategy was independently verified by comparison with direct (non-interpolated) evaluations on selected test cases, showing negligible deviation.

For experimental validation of this approach under nonlinear conditions with single-tone and multi-tone excitations, see, e.g., Roncen & Cardesa (2023) and Roncen (2025*b*). Numerical implementation details, including nonlinear cases, are provided in shared Python codes (Roncen 2025a).

Alternative methods, such as Diab’s model (Diab *et al.* 2022) updated for instantaneous velocity, Moufid’s volume-based approach (Moufid *et al.* 2024), or Monteghetti’s nonlinear oscillo-diffusive method (Monteghetti *et al.* 2018), could yield comparable or better results. The DIR-TDIBC is chosen here primarily for its simplicity of implementation.

### 3.2 Acoustics, turbulence and the DIR-TDIBC

The goal of the DIR-TDIBC method is to produce, from an incident acoustic wave, a reflected wave that will encompass the full acoustic behaviour of the aeroacoustic liner, including its nonlinear effects and its flow effects. A fundamental limitation of the approach, however, is that the incident wave at the liner surface is not directly accessible. Instead, only the wall-normal velocity measured above the liner is available.

In experiments, the wall-normal velocity can be measured using laser Doppler velocimetry (LDV) or time-resolved particle image velocimetry. Similarly, in high-fidelity numerical simulations—where the liner is resolved down to each perforation and no impedance boundary condition is imposed—the velocity can also be extracted directly. In both cases, the concept of homogenised impedance becomes critical: if measurements are taken too close to the liner, the notion of impedance loses its physical meaning. This issue will be further addressed in the discussion of the results.

Whether obtained from experiments or high-fidelity simulations, the evaluated wall-normal velocity signal in the time domain *v*_*n*_ (*t*) contains multiple contributions: the mean flow *v*_0_, hydrodynamic fluctuations related to turbulence *v*_hyd_ (*t*), and acoustic induced perturbations *v*_ac_ (*t*):

(3.4) $$v_{n}(t)=v_{0}+v_{\text{hyd}}(t)+v_{\text{ac}}(t),$$

where we define the fluctuating velocity as

(3.5) $$v^{\prime }=v_{\text{hyd}}(t)+v_{\text{ac}}(t).$$

In the remainder of this work, we also define the mean-removed root-mean-square fluctuating velocity $v_{\text{rms}}^{\prime }$ as

(3.6) $$v_{\text{rms}}^{\prime }=\sqrt{\frac{1}{N_{t}}\sum_{i=1}^{N_{t}}v^{\prime }{\left(t_{i}\right)}^{2}},\;$$

with *N*_*t*_ the number of samples in the time-domain signal.

In the present work, two strong modelling assumptions are introduced, in order to consider a one-way coupling between turbulence and acoustics:

- **Hypothesis 1:** The impedance characterises exclusively the transfer function between acoustic quantities, and turbulent contributions are not directly *modulated* by the impedance. In other words, the divergence-free component of the velocity field—associated with hydrodynamic fluctuations—is assumed to be unaffected by the impedance, whereas the irrotational component—associated with acoustically induced fluctuations—is governed by it.
- **Hypothesis 2:** Turbulence-induced velocity fluctuations contribute to the onset of nonlinear effects described by Eqs. 2.4*a*–2.4*b*. This contribution is accounted for directly in the time domain by updating the impulse response in Eq. 3.1 at each time step, using the total wall-normal velocity *v*_*n*_ (*t*), obtained either experimentally from LDV measurements or numerically from probe data.

Once the total normal velocity above the liner is known, the impedance nonlinear behaviour is therefore fully prescribed in time. An incident wave must then be supplied to the DIR-TDIBC convolution—this corresponds to *x*(*t*) in Eq. 3.2. In a Navier–Stokes characteristic boundary condition (NSCBC) framework, *x*(*t*) = *p*_ac_(*t*)/(*ρ*_*f*_ *c*_*f*_) − *v*_ac_(*t*) corresponds to the incoming acoustic characteristic. During this convolution, the nonlinear response is numerically reconstructed by injecting *v*_*n*_(*t*) in place of ${\tilde{\nu }}_{n}$ in Eq. 3.1, with the impulse response updated at every time step in Eq. 3.2.

The normal velocity *v*_*n*_(*t*) is assumed to be representative of the velocity experienced by a homogeneous impedance boundary condition. This assumption breaks down in the immediate vicinity of the liner, where the impedance is discontinuous due to perforation**–** rigid wall interfaces. Nevertheless, it is assumed that at a sufficient distance above the liner, a homogenised velocity field can be recovered, and that the measured velocity provides a meaningful estimate of the response that would be obtained in an idealised homogeneous configuration.

The above procedure is repeated for each velocity measurement performed above the liner, within a certain distance. This yields the spatial evolution of the impedance as a function of the frequency, over an extended frequency band.

We reiterate that the requirement to use this method is quite strong in terms of modelling, as one already needs to know, through a model such as Eq. 2.3, the proper behaviour of the liner in a linear or nonlinear context. Consequently, the method should be viewed primarily as a tool for assessing the relative effect of flow noise on a liner’s impedance. Nevertheless, provided that the frequency-domain models employed are accurate, which is expected for well-studied liners such as SDoF liners, the resulting impedance estimates are expected to be close to the true values.

### 3.3 Signal processing procedure

The velocity signals obtained from the LDV measurements and the LBM-VLES simulations were processed using similar workflows, with a few case-specific differences. The LDV post-processing is described first, followed by the modifications applied to the LBM-VLES data.

For each measurement location, the wall-normal velocity signal was interpolated onto a uniformly sampled time grid using linear interpolation over the ≈ 10 s of acquisition. The resulting signal was divided into overlapping segments of 1s in duration (approximately 1400 acoustic periods), with a 50% overlap between consecutive segments.

Each segment was supplied to the DIR-TDIBC framework, and the reflected acoustic wave was reconstructed through the dynamic convolution procedure described in Section 3.1. The resulting time-domain signal was multiplied by a Hanning window prior to Fourier transformation. The reflection coefficient was then extracted at the acoustic excitation frequency and converted into impedance using Eq. 2.9.

For the LES, only a single 11.4 ms realization (16 acoustic periods) was available due to the computational cost of the high-fidelity simulation. No temporal interpolation was required since the time step was fixed.

## 4 Experimental and numerical configurations

The methodology developed in this work is applied to two distinct datasets:

- experimental measurements performed in the ONERA B2A wind-tunnel using LDV velocity measurements above an acoustic liner (ONERA sample), whose analysis is detailed in Section 5, and
- high-fidelity numerical simulations of turbulent flow over a perforated liner using an LBM–VLES approach carried out at PoliTo (PoliTo sample), whose analysis is detailed in Section 6.

Although both configurations involve grazing flow over an SDoF liner and are analysed using the same impedance-eduction methodology, they were originally developed in separate studies and were not designed as a strict one-to-one comparison case.

Nevertheless, the two configurations share many important features. In both cases, the liner consists of a perforated facesheet covering an array of square cavities, and the acoustic response of the liner is investigated under turbulent-grazing-flow conditions with a harmonic acoustic excitation. The flow Mach numbers, excitation frequencies, and general geometric scales are also comparable. This similarity makes it possible to apply the same analysis framework to both datasets and to examine how the proposed methodology behaves in two complementary contexts: a controlled experimental environment and a fully resolved numerical dataset where the reference impedance can be directly evaluated.

At the same time, several differences must be emphasised. The experimental configuration corresponds to the B2A facility geometry and liner sample, whereas the numerical simulation reproduces a liner test rig geometry inspired by the UFSC facility (Quintino *et al.* 2025). As a result, some geometric parameters differ, including perforation diameter and global porosity. The acoustic excitation frequency and the accessible measurement locations are also not identical. In addition, the numerical simulation employs a reduced spanwise domain with periodic boundary conditions, which constrains the development of large turbulent structures compared with the experimental facility. Additional information on the measurement methodology, velocity field and spectral content is provided by Ambrosiani *et al.* (2026). The reliability of the numerical results is supported by a dedicated mesh-convergence analysis and comparison with experiments reported by Paduano *et al.* (2026), while a more comprehensive characterisation of the velocity field and its energy content is provided by Scarano *et al.* (2026).

Consequently, the purpose of presenting both datasets is not to perform a direct quantitative validation of the numerical simulation against the experiment. Instead, they should be viewed as two representative configurations that allow the proposed methodology to be evaluated under realistic conditions. The experimental dataset provides insight into the behaviour of the method with real measurements affected by noise, limited spatial resolution, and experimental uncertainties. The numerical dataset, on the other hand, offers a controlled environment in which the true impedance can be determined independently, enabling a more direct assessment of the method’s accuracy.

**Figure 3:**
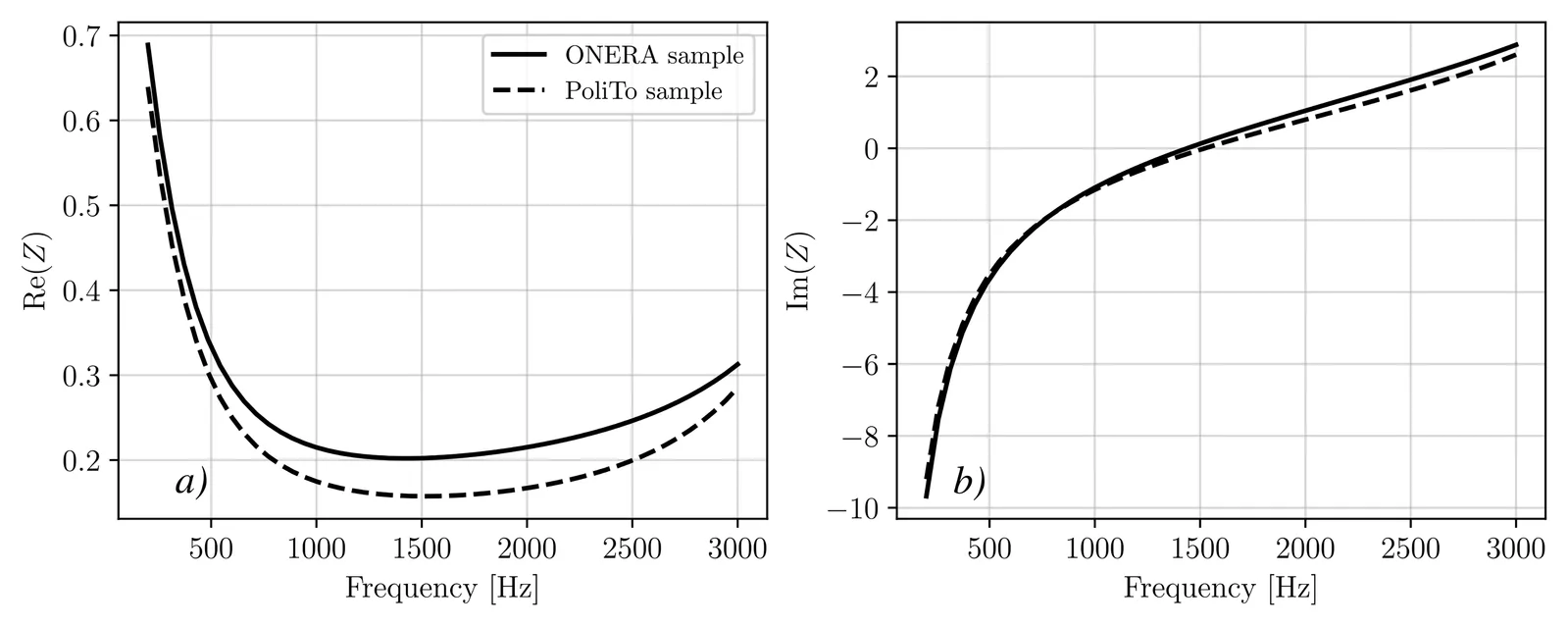
Predicted linear impedance of the ONERA and PoliTo samples. a) Resistance. b) Reactance.

**Figure 4:**
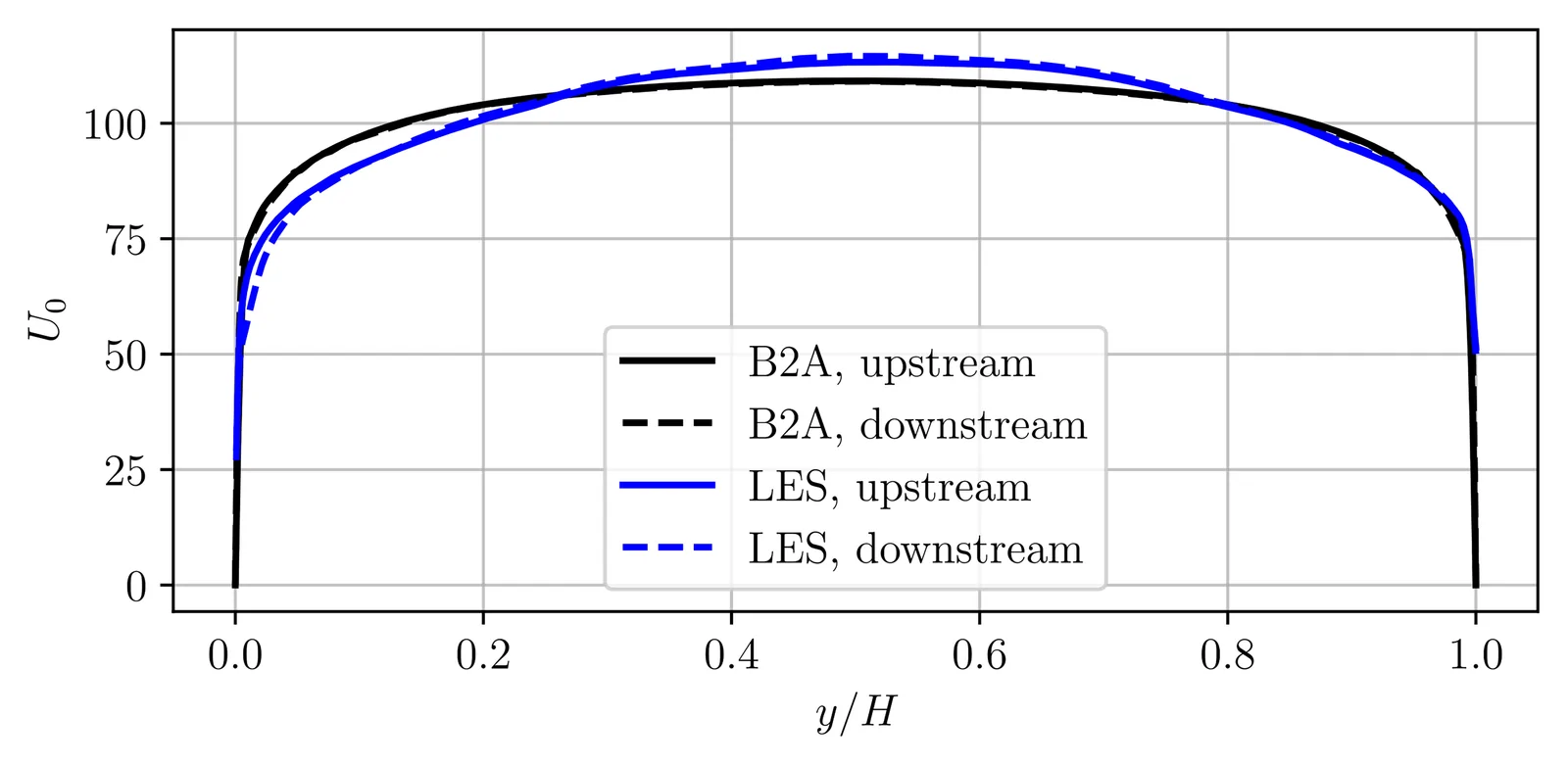
Comparison of flow profiles in the B2A duct (obtained via LDV) and in the LES carried out at PoliTo, evaluated in all cases at the same spanwise location. In the LDV case, the measurements are taken 3mm away from the liner, while in the LES case, the evaluation is exactly at the liner beginning and end.The experimental profiles are extended to *y* = 0 by imposing the wall boundary condition *U*_0_ (0) = 0, although the LDV measurements are available only for *y*/*H* ≳ 0.04.

The main characteristics of the experimental and numerical configurations are summarised in Table 1, which highlights both their similarities and their differences. The predicted linear impedance of each sample, obtained from Eq. 2.2, is shown in figure 3. The flow profiles measured without acoustic excitation just upstream and downstream of the liner samples are shown in figure 4. The following sections then describe the experimental set-up and the numerical methodology in detail before presenting the corresponding results.

## 5 Application of the DIR-TDIBC to LDV measurements

This section introduces the This section introduces the experimental set-up used at ONERA for the liner characterization setup used at ONERA for the liner characterization under shear-grazing-flow conditions. Section 5.1 presents the B2A wind tunnel facility, Section 5.2 the LDV set-up, and Section 5.3 the liner sample used in this work. The configurations under study are detailed in Section 5.4, and results of the DIR-TDIBC analyses are presented in Section 5.5.

### 5.1 The B2A wind-tunnel

**Figure 5:**
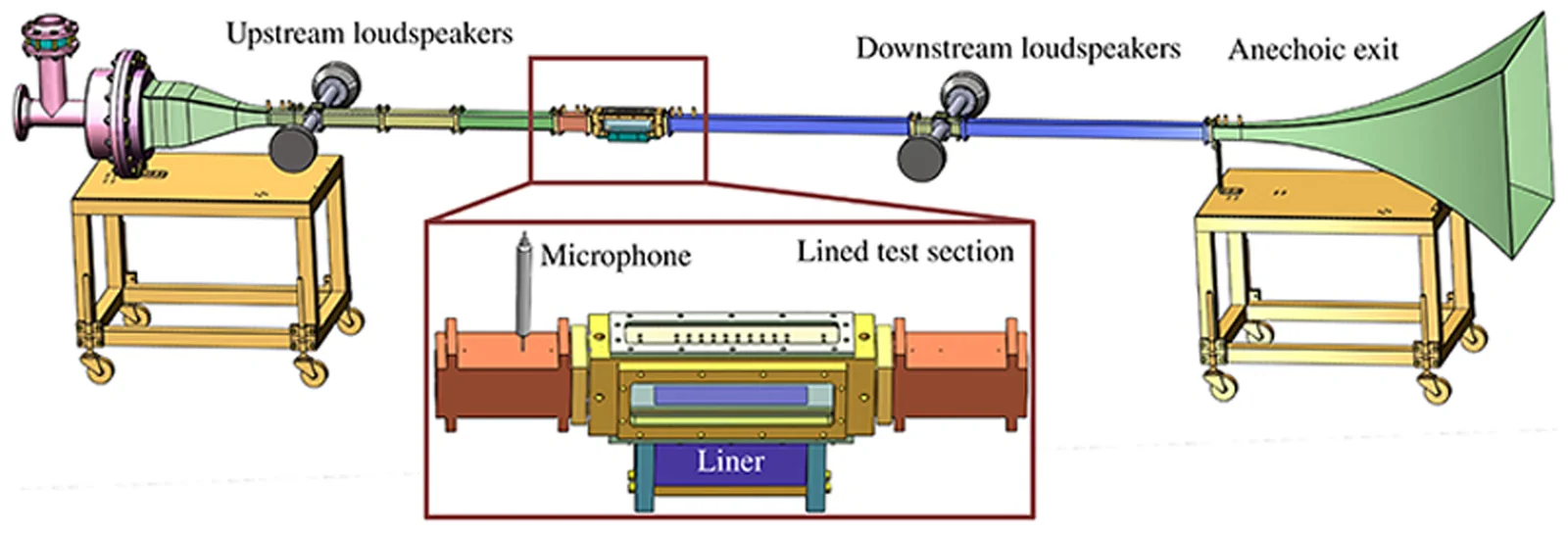
The B2A facility located at ONERA Toulouse.

**Figure 6:**
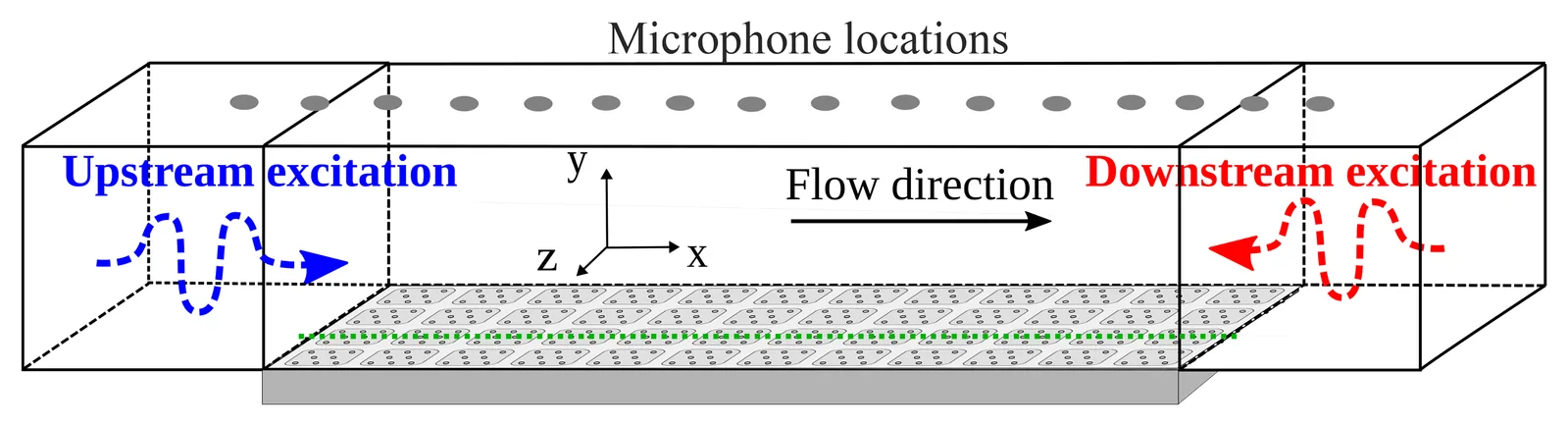
Schematics of the LDV measurement and microphone locations. Upstream and downstream excitations are never on simultaneously. The green line represents the spanwise location of LDV measurements and can be located at different heights above the liner sample.

The ONERA B2A facility consists of a 4-m-long stainless-steel duct with a square cross-section of 50 × 50 mm. The 0.2-m long test section is equipped with silica windows for optical access, and the duct termination features a quasi-anechoic outlet, yielding a downstream reflection coefficient below 0.2 for frequencies above 500 Hz. The facility, shown in figure 5 can generate fully developed turbulent flows with bulk Mach numbers up to *M*_b_ = 0.6, where the turbulence intensity in the test-section centerline is of the order of a few percents. Two acoustic drivers located upstream or downstream of the test section can produce multi-tone signals of up to 150 dB over the frequency range 300**–**3 500 Hz. The test liner occupies a 150-mm-long segment of the lower duct wall. Six microphone locations are available on the upper wall to measure the acoustic pressure field upstream of the liner leading edge and downstream of the liner trailing edge. The upper wall facing the liner is equipped with 16 microphone locations ranging from *x* = − 27 mm to *x* = 177 mm from the liner origin with a step of 12 mm between each locations as represented in figure 6. Two flush-mounted GRAS microphonic probes are used (probe diameter of 1.2 mm) to set the incident upstream or downstream acoustic excitation and to measure the SPL at the wall opposite the liner via a wave-sorting procedure (Lafont *et al.* 2020). A single microphone is then used at each measurement location to retrieve the pressure signal, without phase mismatch between measurements.

### 5.2 The LDV set-up

A two-component fringe-mode LDV system enables measurement of both axial and vertical velocity components across nearly the entire volume of the test section, using a displacement bench. This measurement system was composed of a laser emitting green (514.5nm) and blue (488nm) wavelengths. The two pairs of beams were issued by a DANTEC 55X emitting head equipped with a 240mm focal lens. The fringe spacing for the green beam is 2.2344 *μ*m and the fringe spacing for the blue beam is 2.1544 *μ*m. The crossing angles are measured and used as an input for the post-processing of the signals. The transformation matrix has an accuracy estimated to be lower than 1%. Signals were processed by a DANTEC BSA burst spectrum analyser. The emitting optics produce an elliptical measurement volume whose minor axis can be as small as 70 *μ*m and the major axis is about 0.7 mm.

Flow is seeded with amorphous silica particles, chosen for their low tendency to deposit on optical windows. Because particles arrive randomly in the measurement volume, the LDV signal is unevenly sampled; a reconstruction algorithm is used to resample the raw data at a uniform rate by the use of a linear interpolation in time. Each velocity component is measured at a minimum rate of *f*_*m*_ ≈ 15 000 samples per second with a 97% validation rate in coincidence, with more than 250 000 total samples acquired at each measurement location.

The experiments were conducted under standard atmospheric conditions, with the flow temperature maintained at ambient *T*_0_ ≈ 20 °C, with maximum fluctuations of 1 °C, corresponding to a sound speed of *c*_0_ ≈ 343 m /s. The mass flow rate was maintained constant at either 100 g/s or 300 g/s for all experiments, corresponding to a bulk Mach number of either *M*_b_ = 0.1 or *M*_b_ = 0.3.

**Figure 7:**
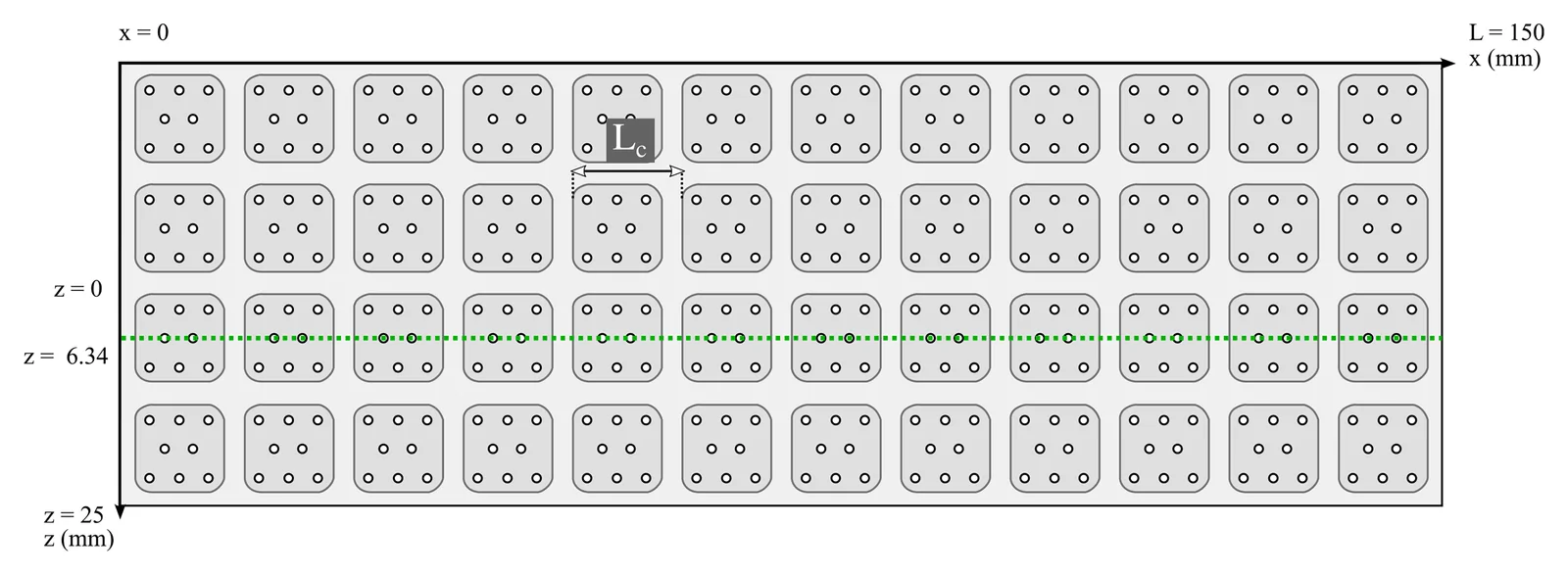
Top view sketch of the liner SDoF sample. The green line represents the spanwise position of the LDV measurement line and is located at different heights above the liner sample. Length ***L***_*c*_ = 12.44mm represents the inter-cell distance.

The resulting LDV dataset comprises over 120 spatial measurement points per tested configuration. The system provides two-dimensional velocity fields in the *x* line at *y* = 1mm or *y* = 2.5mm from the liner surface and *z* = 6.34mm away from the centre of the sample, centred in the middle of a row of cavities, as shown by the green dashed line in figures 6-7. Only the streamwise and vertical (normal to the liner) components are measured.

### 5.3 Liner sample

The acoustic liner used for this study is an SDoF liner made of a perforated plate over squared cavities based on a geometry shared between multiple facilities (Quintino *et al.* 2025). Each cavity is covered by a thin plate of 0.635 mm with eight orifices of diameter *d* = 0.991 mm for a total porosity of *ϕ*_*p*_ = 3.95%. The cavity height is *H*_*c*_ = 38.1 mm, with a side length of *D* = 9.91 mm large. A top-view sketch is given in figure 7, with the LDV line super-imposed to give its location over the liner.

**Figure 8:**
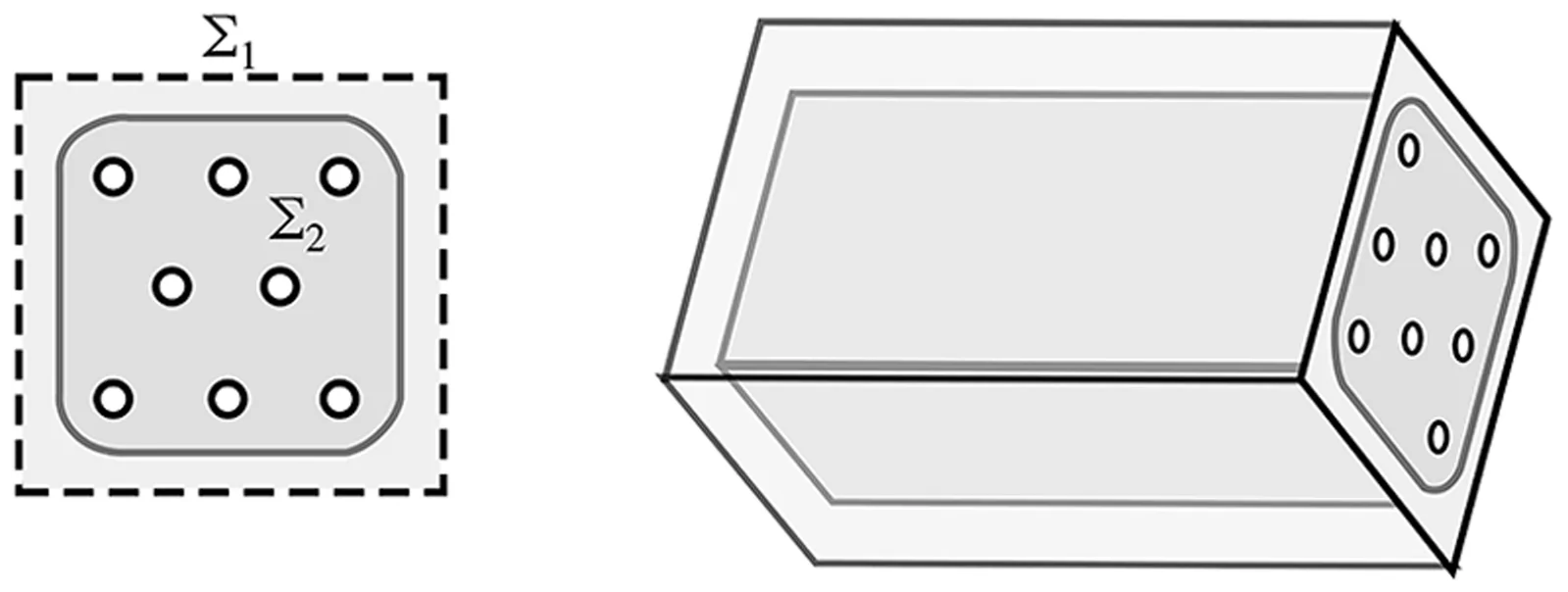
Top-view and side-view perspective of a liner cell.

Before presenting the results, a particular feature of the tested liner sample must be discussed. The sample is composed of *N* elementary cells, separated by thick walls – whose half thickness *w*_*p*_ is given in table 1– which account for approximately 37% of the liner surface area, as represented in figure 8. Using the notations in the figure, *Σ*_2_/*Σ*_1_ = 0.63.

When considering the cell as a whole (*Σ*_1_), the global porosity is 3.95%. However, if one examines only the perforated regions of each acoustically active cell, excluding the cavity walls, the effective (local) porosity based on *Σ*_2_ rises to 6.5%.

To correctly represent the impedance under this interpretation, the liner can be modeled as a dual-liner composed of two distinct cell types assembled in parallel: rigid walls in *Σ*_1_\*Σ*_2_ and single-degree-of-freedom (SDoF) resonators with a porosity of 6.5% in *Σ*_2_. This modelling choice has implications for the predicted nonlinear behaviour of the liner, since nonlinear effects are directly influenced by the local porosity; see Eqs. 2.4*a* and 2.4*b*. Overall, our choice seems to be aligned with the recent conclusions of Jones & Nark (2023).

### 5.4 Configurations under study

Measurements were performed during an LDV experiment at bulk Mach numbers of 0.1 and 0.3, with an acoustic excitation at 1500 Hz ranging from 115dB to 145dB (incident SPL at the entrance of the test section). The experiment was performed with both loudspeakers being either upstream or downstream of the liner section. The methodology described in Section 3.2 is performed on each of the ≈ 120 LDV measurement points located on a line 1mm above the liner (see figure 6).

Table 2 summarises the configurations under study.

### 5.5 Results

We first characterise in Section 5.5.1 the combined flow and acoustic environment around the liner. The flow and acoustics characterization highlights the relative amplitudes and frequency content of the flow and acoustic contributions using LDV measurements. Second, the spatial evolution of the impedance shows the local variation of resistance and reactance between the measurement points for different SPLs and flow conditions using the DIR-TDIBC in Section 5.5.2.

These local results are then synthesised into a single effective impedance value through an admittance averaging procedure in Section 5.5.3. Finally, this global impedance provides the framework for Section 5.5.4, where results from a linearised harmonic solver are validated against experimental microphone pressure measurements.

#### 5.5.1 Flow and fluctuation characterization

**Figure 9:**
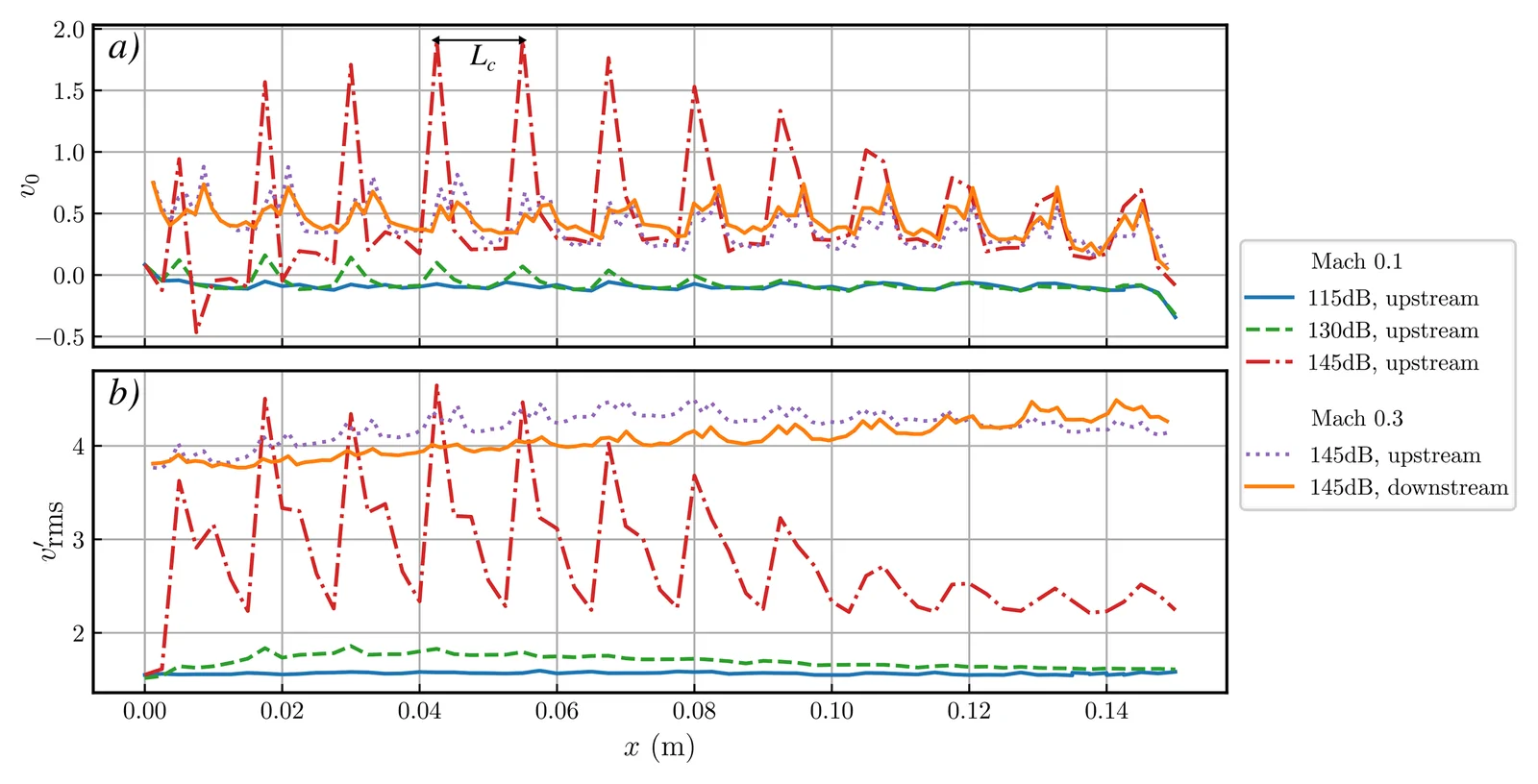
a) Mean and b) mean-removed rms components of the wall-normal velocity field above the liner at a distance of 1 mm. Length *L_c_* denotes the inter-cell distance, as represented in figure 7.

The streamwise evolution of the mean wall-normal velocity component *v*_0_ along the liner is shown in figure 9 for the different configurations, highlighting the substantial influence of SPL on the mean flow. The measured values remain close to zero over the rigid part of the liner for most of the configurations, i.e., not exceeding on average 1% of the mean flow streamwise velocity, indicating that the LDV beams alignment does not introduce a significant bias in the wall-normal direction measurement. Local variations observed at specific streamwise locations coincide with the positions of the liner orifices, suggesting a strong wall-normal bias flow induced by the acoustic forcing in the orifices. The third configuration, i.e. that with the lowest mean Mach number and the highest SPL, provides a different trend in which the competitive effect between the flow and the acoustic is fully dominated by the acoustic and therefore increases *v*_0_ along the liner with strong local variations in the orifices.

The streamwise distribution of the (mean-removed) wall-normal velocity fluctuations, quantified by the root-mean-square value $v_{rms}^{\prime }(x)$ (*x*), is also shown in figure 9. The fluctuation level exhibits a clear streamwise evolution, reflecting the development of the grazing turbulent boundary layer in configurations 4 and 5, similarly to what is described by Paduano *et al.* (2026). The local variations are correlated with the location of the orifices and show a strong increase in vertical velocity fluctuation over the liner, attributed to the acoustic contribution. The progressive attenuation of the amplitude of the local peaks is attributed to the acoustic attenuation brought by the liner.

#### 5.5.2 The DIR-TDIBC prediction: spatial evolution of the impedance

**Figure 10:**
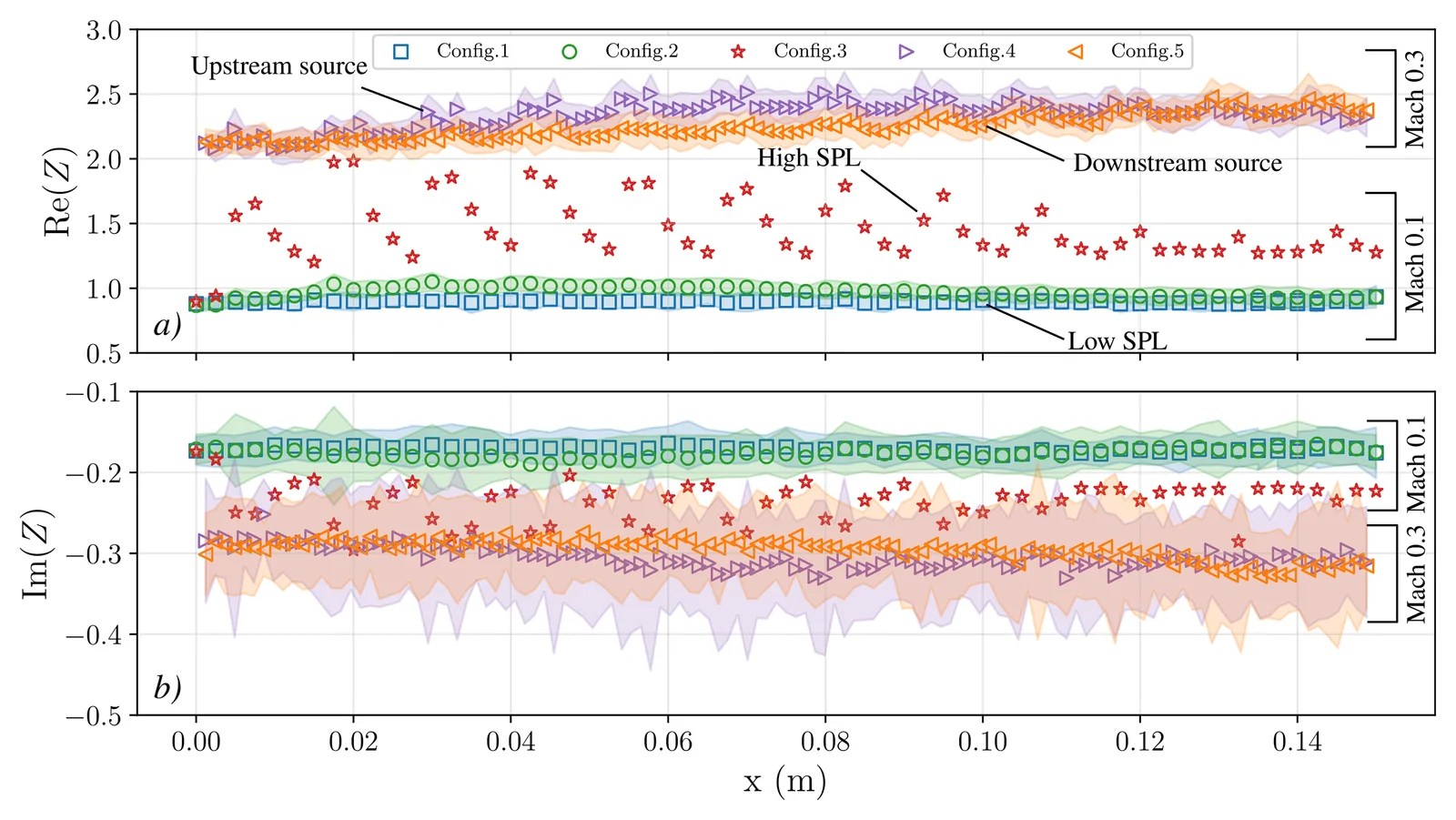
The DIR-TDIBC-based eduction using LDV measurements. Comparison of the impedance at 1500Hz as a function of the streamwise direction, for the different configurations described in table 2. Coloured areas correspond to ±3 standard deviations. a) Resistance. b) Reactance.

Impedance results obtained at an excitation frequency of 1500 Hz with the DIR-TDIBC methodology presented in Sec. 3.1, are shown at each of the LDV measurement points in figure 10 for all configurations. Due to the long duration of LDV acquisition at each location (≈ 10 s), the DIR-TDIBC method was applied on overlapping time windows of approximately 1 s each, with 50% overlap. The DIR-TDIBC requires approximately 1 s per case when implemented in the Julia language (Bezanson *et al.* 2017) using just-in-time compilation, suggesting that real-time impedance assessment could be attempted in future tests.

The impedances at location *x*_*i*_ shown in figure 10 therefore correspond to the average over *N*_seg_ ≈ 20 time segments, i.e.,

(5.1) $$Z_{x_{i}}=\frac{1}{N_{\text{seg}}}\sum_{n=1}^{N_{\text{seg}}}Z_{x_{i}}^{\left(t_{n}\right)}.$$

Some clear trends can be observed for the resistance and reactance. Among these trends, resistance increases with higher SPL at Mach 0.1, and also increases when, for the same SPL, the flow Mach number is raised to 0.3, consistent with previous observations in the literature. Similarly, the reactance decreases when the SPL or Mach number is increased.

At Mach 0.1 and for an excitation of 115dB, the impedance does not vary spatially, indicating that nonlinear effects are negligible in this case. When increasing the SPL to 130dB, a slight increase in the resistance is observed in the first few centimeres of the liner, before this effect cancels near the end of the liner, rejoining the impedance obtained at 115dB. This marks the onset of nonlinear effects.

A striking feature appears in the resistance data at Mach 0.1 and SPL 145 dB (configuration 3, red stars in figure 10), exhibiting strong spatial oscillations that are absent in the other configurations. These oscillations are attributed to the onset of vortex shedding in the liner orifices, induced by nonlinear effects at high SPL, which explains their absence in other Mach 0.1 cases of lower SPL. The oscillation period is 1.25 cm, corresponding exactly to the size of a single liner cell. The observed peaks align precisely with the orifice locations along which the LDV measurement line is taken, as illustrated in figure 7.

**Figure 11:**
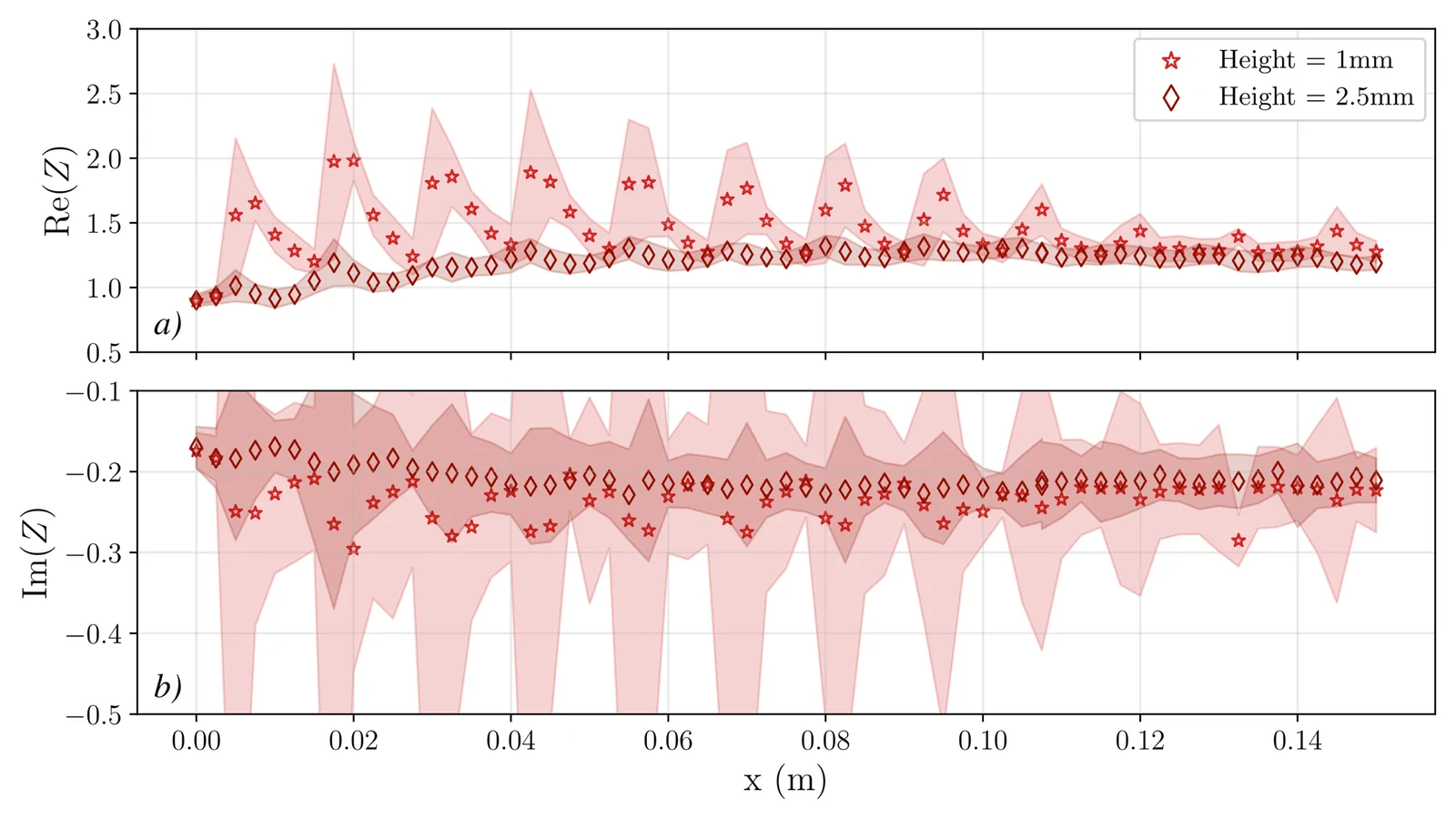
The DIR-TDIBC-based eduction using LDV measurements. Comparison of the impedance at 1500Hz as a function of the streamwise direction, at Mach 0.1 and with an incident SPL of 145dB (configuration 3), for two different heights of the LDV line above the liner. Coloured areas correspond to ± 3 standard deviations. a) Resistance. b) Reactance.

The oscillations at Mach 0.1 and SPL of 145 dB are reduced near the end of the liner due to the lower SPL resulting from liner absorption. Additional measurements taken further above from the liner confirmed a smoothing of these features, as shown in figure 11 for measurements performed at a distance of 2.5mm above the liner. This represents a limit case where the present method may be less suited. Indeed, one expects the impedance to be high near the rigid wall and lower near the perforations. However, the DIR-TDIBC method enforces a homogeneous impedance representation and interprets a rigid wall—i.e., a region with low velocity fluctuations—as a less nonlinear portion of the liner. Measurements performed at a greater distance from the liner inevitably contain less local information about the near-wall flow properties, and the level of accuracy that can be expected from the proposed approach in this case is not straightforward to assess. Increasing the distance from the liner tends to reduce the influence of local discontinuities and may therefore improve the validity of a homogenised impedance representation. However, this comes at the cost of a potential loss of accuracy in the impedance evaluation, which remains to be quantified.

To begin the assessment of this trade-off, companion simulations were performed in a simplified two-dimensional grazing configuration without mean flow, in order to isolate the loss of accuracy caused by purely acoustic, non-viscous effects when the impedance is “measured” at increasing distances from the liner surface. At 1500 Hz, these simulations indicate (see Appendix A for details) that the evaluated impedance remains within 0.1 of the true value when the measurement is taken 2.5 mm above the liner, for a target value of 0.8 in this case. Although this estimate is only indicative, it suggests that, in the present configuration, measuring at this height represents a reasonable compromise when measurements closer to the liner are biased by non-homogenisable effects.

More generally, the interplay among impedance homogenization, acoustic propagation, and turbulent fluctuations deserves further investigation and will be addressed in future work, with some of these ideas already introduced in Appendix A in order to give rough guidelines for LDV height selection, rooting the analysis in a simplified jet-in-crossflow model.

At Mach 0.3 and SPL of 145 dB, the oscillations observed at Mach 0.1 are still visible but strongly damped, likely because the flow eddies overshadow SPL-induced vortex shedding and shear enhances mixing, producing a smoother resistance profile. This illustrates the competitive effect between flow and SPL described by Léon *et al*. (2019).

We initially expected the resistance to decrease and the reactance to increase along the liner in the direction of wave propagation, due to the reduction in SPL as the liner absorbs the wave. Instead, at Mach 0.3, resistance increases and reactance decreases along the *flow direction*, regardless of the acoustic source location. This behaviour matches expectations for the downstream-source experiment, but not for the upstream-source one. This finding contradicts our previous work, where the nonlinear behaviour of the liner, governed by SPL only, suggested a decrease in resistance along the liner in the incident wave direction (Lafont *et al.* 2020; Roncen *et al.* 2022).

The difference may be due to increased turbulence over the liner contributing more strongly to the impedance, and to mean flow effects associated with the shear-layer development above the liner (Paduano *et al.* 2026). Remarkably, this behaviour is observed irrespective of the acoustic source location. The spatial evolution of the educed impedance is essentially identical for both upstream- and downstream-source configurations. This indicates that, under the present conditions, the variation of resistance and reactance along the liner is not primarily governed by the direction of acoustic-wave propagation. Instead, it appears to follow the streamwise development of the grazing flow and the associated turbulence over the lined surface. In the experiments of Lafont *et al*. (2020) and Roncen *et al.* (2022), the Mach number was limited to 0.1, which may explain why good agreement was still found with a spatially decreasing resistance: the turbulent flow noise may not have been strong enough to significantly affect the educed impedance, similarly to the present experiments at Mach 0.1.

We conclude that antagonistic effects of flow and acoustics appear in the upstream-source case, where the flow turbulence increases the resistance and decreases the reactance along the flow direction, while the reduction of SPL in the flow direction decreases the resistance and increases the reactance. In the downstream-source case, both effects contribute similarly to the changes in resistance and reactance.

#### 5.5.3 Admittance-averaged global impedance

To obtain a single effective impedance value *Z*_mean_ from all *N*_*x*_ LDV measurement locations, we first compute the admittance at each location, *Y*_*n*_ = 1 /*Z_n_*, and then perform an ensemble average over time segments. Specifically, at each location *x*_*n*_, the impedance $Z_{x_{n}}^{\left(t_{j}\right)}$ was evaluated via the DIR-TDIBC on multiple overlapping time windows *t*_*j*_, *j* = 1, …, *N*_seg_. A Monte Carlo approach is used: for each of *N*_MC_ = 5 000 iterations, one impedance value is randomly drawn from the available segments at each location, and the corresponding admittances are averaged over all locations. The mean impedance is then obtained as

(5.2a) $$Y_{\text{mean}\;}=\frac{1}{N_{\text{MC}}}\sum_{k=1}^{N_{\text{MC}}}\frac{1}{N_{x}}\sum_{n=1}^{N_{x}}\frac{1}{Z_{x_{n}}^{\left(t_{k,n}\right)}},$$

(5.2b) $$Z_{\text{mean}\;}=\frac{1}{Y_{\text{mean}\;}},$$

where *t*_*k,n*_ is randomly selected among the *N*_seg_ time segments available at location *x*_*n*_ during the *k*-th Monte Carlo iteration.

This approach is equivalent to treating the liner as a parallel assembly of non-communicating cells, each having their own impedance values, a phenomenon used to create acoustic meta-surfaces. Once the mean admittance is calculated, the resulting impedance is considered to be the effective global impedance of the liner, close to what a classical impedance eduction approach would “see”.

**Figure 12:**
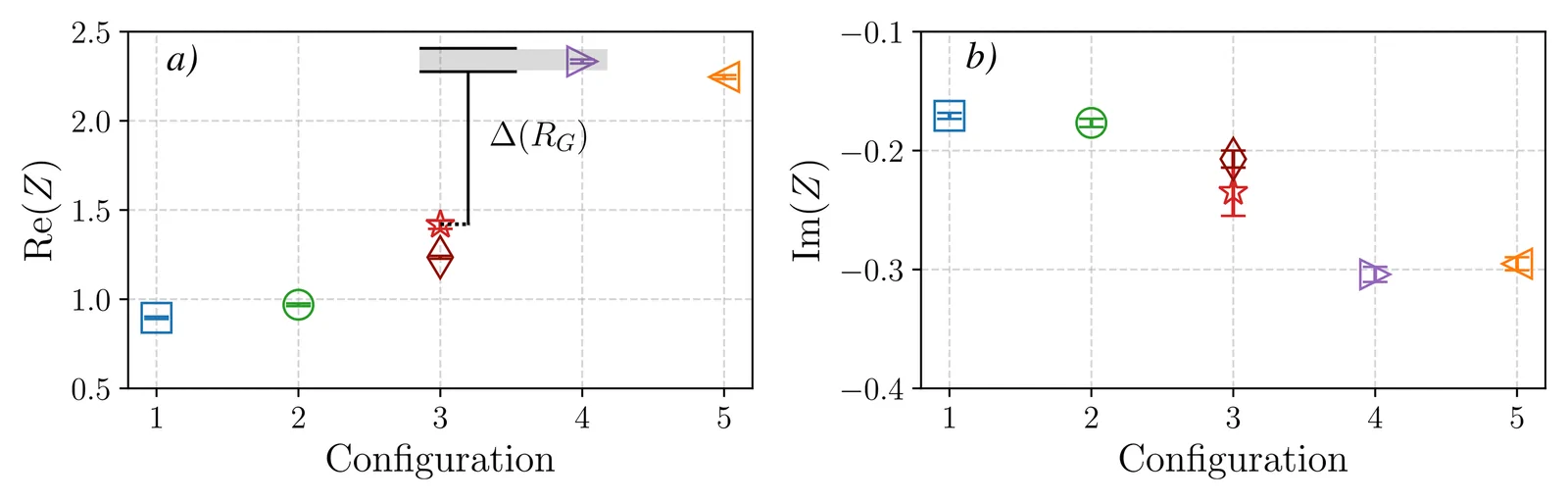
(Colour online) Comparison of the spatially averaged impedance for the configurations described in table 2. For configuration 3, the different symbols correspond to the LDV measurement distance from the liner, as shown in figure 11. Here *Δ R*_*G*_ corresponds to the prediction of the Goodrich model given by Eq. 2.7 to compare configurations 3 and 4. a) Resistance. b) Reactance.

In practice, this averaging has limitations, particularly at higher frequencies, where the homogenization assumption becomes less valid. Nevertheless, the average impedance is used for comparison between configurations in figure 12, with error bars representing three standard deviations computed from the Monte Carlo procedure.

The classical behaviour is observed: resistance increases and reactance decreases with higher SPLs or in the presence of shear grazing flow. In this analysis, however, no explicit model for the flow effect was used; it was captured only indirectly through its turbulent noise contribution.

Using the Goodrich model of Eq. 2.7, it is possible to predict the change in resistance Δ (*R*_*G*_) between the Mach 0.3 and Mach 0.1 configurations at comparable SPL and comparable source location. This estimate assumes that the displacement boundary-layer thickness *δ*^★^ remains unchanged between the two cases. In the present experiments, equation 2.8 with LDV measurements taken on a wall-normal profile yielded *δ*^★^ = 2 ± 0.3 mm, a value consistent with those reported by Yu *et al.* (2008) for the NASA Langley grazing-incidence tube, which features a cross-section geometry similar to that of the B2A bench.

Comparing configurations 3 and 4, which have a different Mach number but a similar SPL, the Goodrich model predicts Δ (*R*_*G*_) ∈ [0.94, 1.12], which is in close agreement with the values obtained using the DIR-TDIBC approach, as shown in figure 12.

#### 5.5.4 Comparison of predictions with pressure microphone measurements

The accuracy of the educed impedances is assessed by comparing numerical predictions with experimental acoustic pressure measurements. The linearised Euler equations (LEEs) are solved in a two-dimensional domain using a discontinuous Galerkin (DG) method to simulate the acoustic propagation within the B2A test section, in the frequency domain. The liner’s impedance is taken as that obtained via the DIR-TDIBC method (spatially averaged based on the admittance of Sec. 5.5.3), i.e., 2.33 −0.30j and 2.25− 0.30j, for configurations 4 and 5, respectively.

Numerical simulations are performed for the Mach 0.3 configurations (configurations 4 and 5). The acoustic pressure is evaluated along the upper wall of the duct, opposite to the liner, to match the locations of the wall-mounted microphones used during the experimental campaign obtained in the B2A facility at ONERA from microphones mounted on the wall opposite the liner, 5 cm above the liner surface (see figure 6 and the description of microphone location in Sec. 5.1).

Here the acoustic contribution is extracted from the pressure microphone measurement via a coherence-based rejection algorithm. In the LEE setup, the shear flow profile of Rienstra & Vilenski (2008, Eq. 4) is taken, with a shear parameter of 0.1. The flow profile is adjusted to match the bulk velocity of the three-dimensional experiment.

**Figure 13:**
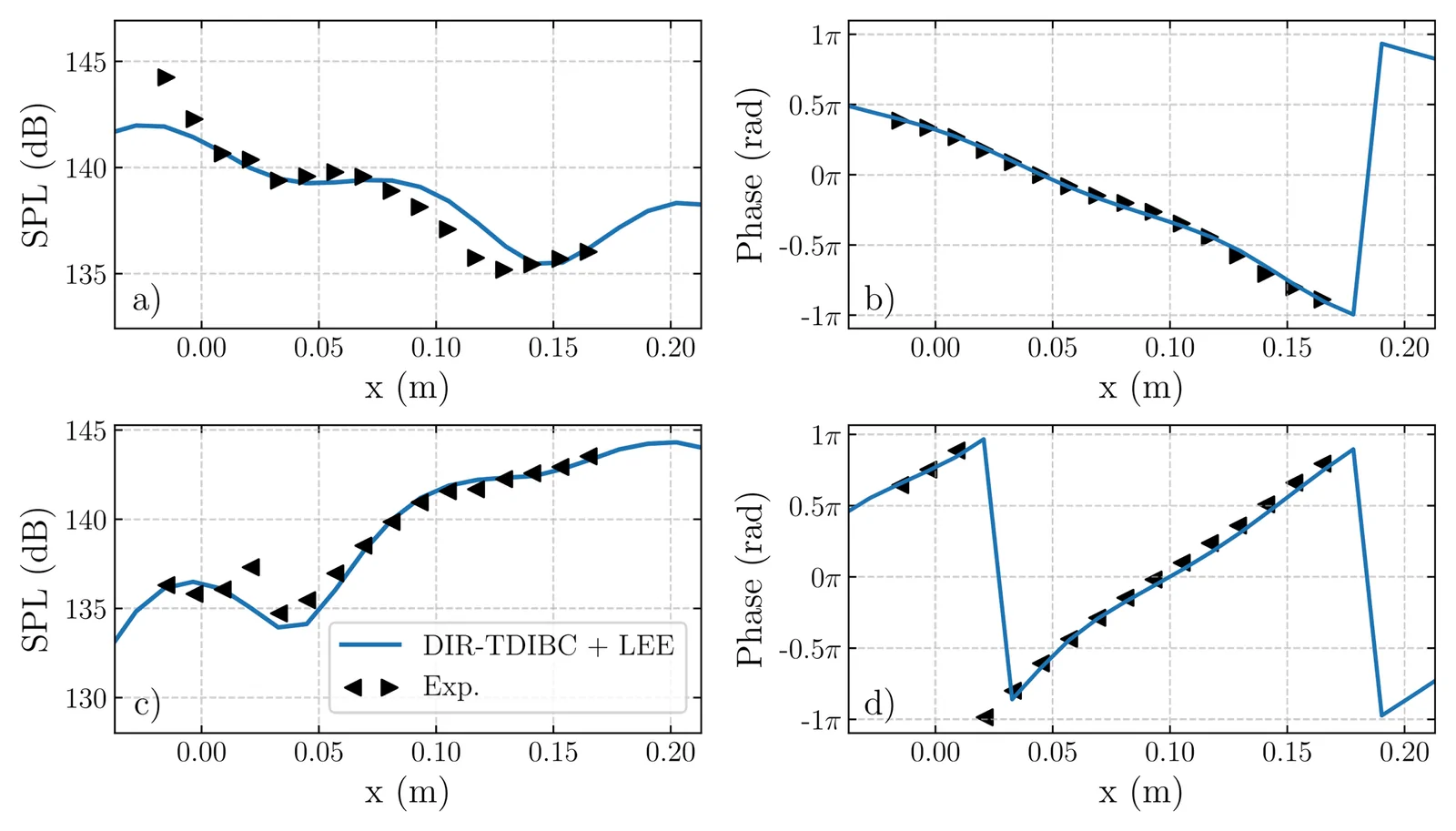
Comparison of acoustic pressure — (a, c) SPL and (b, d) phase — between experimental microphone measurements in the B2A facility at Mach 0.3 (symbols) and numerical predictions from a two-dimensional LEE solver (lines), using the impedance stemming from the DIR-TDIBC approach of Section 5.5.2. (a, b) Configuration 4. (c, d) Configuration 5. Flow goes from left to right. Liner located between *x* = 0.437m and *x* = 0.587m.

The results, presented in Figure 13, demonstrate correct agreement between the LEE predictions and experimental data.

This comparison should not be interpreted as a validation of the nonlinear impedance model itself, but rather as a consistency check of the effective impedance obtained through the DIR-TDIBC procedure. Indeed, once the impedance is identified (including nonlinear and flow-induced effects), the subsequent acoustic propagation problem remains linear, and the LEE framework provides a standard benchmark for assessing whether the resulting boundary condition reproduces the measured pressure field.

#### 5.5.5 Direct eduction approach

To provide a comparison with the previous DIR-TDIBC results, the microphone pressure measurements were also analysed using a classical direct eduction approach based on the KT algorithm (Watson *et al.* 2015), in the frequency domain. This method fits a wavenumber to the pressure measurements directly above the liner and uses the resulting relationship between the wavenumber and the impedance to retrieve *Z*, assuming a homogeneous mean flow profile. This is the approach used in the seminal work of Renou & Aurégan (2011), who first highlighted the upstream-downstream impedance mismatch.

The cases considered are those at Mach 0.3, with both upstream and downstream excitation cases, configurations 4 and 5 in Table 2, respectively.

**Figure 14:**
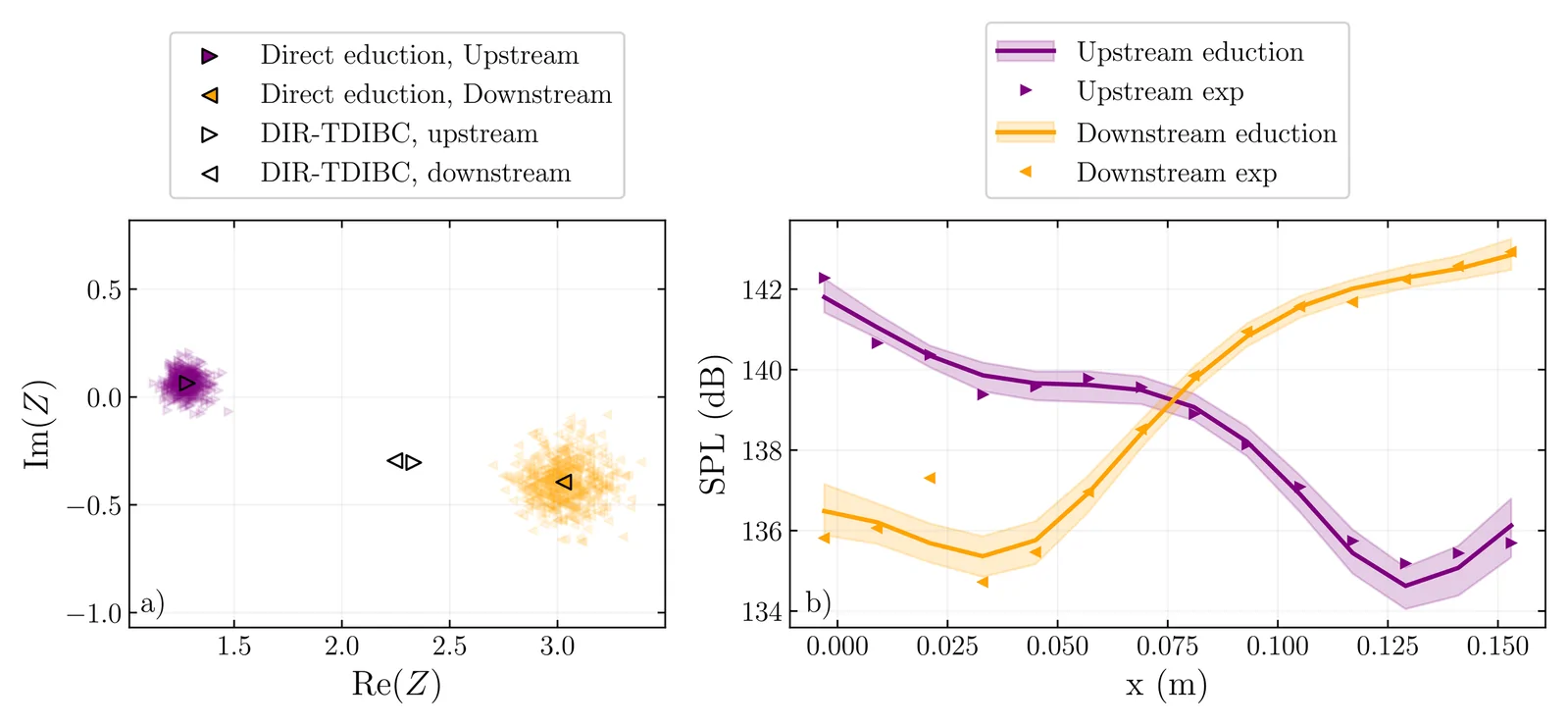
Results of the direct eduction approach at Mach 0.3 with an excitation frequency of 1500 Hz, based on pressure microphones located on the wall opposite the liner. (a) Impedances in the complex plane for each sample and their mean, as well as the mean impedance obtained via DIR-TDIBC in figure 12 for configurations 4 and 5. (b) Corresponding comparison of measured and predicted SPL above the liner. The shaded envelope denotes the minimum and maximum limits across samples. Flow goes from left to right. Liner located between *x* = 0m and *x* = 0.15*m*.

To quantify uncertainty in the eduction process, the SPL and phase at the microphone locations were assumed to have standard deviations of 0.1 dB and 0.1 degree, respectively, consistent with typical microphone measurement accuracy. A Monte Carlo procedure was then performed with 500 realisations to propagate these uncertainties through the inversion process. The resulting impedances and SPL fits are shown in figure 14. A notable observation is that, despite good agreement between measured and modeled pressures, the retrieved impedances exhibit significant differences between the upstream and downstream configurations.

The impedance obtained in the upstream configuration is 1.28 + 0.06j, whereas the downstream configuration yields 3.02 − 0.40j. Using the impedances obtained with the DIR-TDIBC for cases 4 and 5 (2.33− 0.30j and 2.25 − 0.30j, respectively), we observe in figure 14 that the resulting values lie almost exactly on the line connecting the upstream and downstream impedances obtained with the KT algorithm.

## 6 Application of the DIR-TDIBC to LES data

A high-fidelity simulation of turbulent flow over a liner was performed and is analysed in this section to assess the performance of the developed methods on a dataset where the true impedance is precisely known. The numerical approach is recalled in Section 6.1, and the configuration under study is detailed in Section 6.2, with a characterization of the flow and acoustic features provided in Section 6.3. The liner impedance is then evaluated using the DIR-TDIBC approach in Section 6.4, and the results are compared with the exact reference values.

### 6.1 The LBM–VLES method

The high-fidelity simulations are performed with PowerFLOW^©^ (version 6), which is based on the Lattice Boltzmann Method (LBM). A detailed introduction to the LBM is provided by Succi (2001). In the LBM framework, the fluid is represented at a mesoscopic level through particle distribution functions, whose moments recover macroscopic quantities such as density and momentum.

The method can be related to the continuous Boltzmann equation:

(6.1) $$\frac{\partial g}{\partial t}+\xi \cdot \nabla_{x}g+F\cdot \nabla_{\xi }g=\Omega (g),$$

where *g* (***x***, ***ξ***, *t*) is the particle distribution function, ***ξ*** is the microscopic velocity, ***F*** denotes external/body forces, and *Ω*(·) is the collision operator. Following common practice, the Bhatnagar–Gross–Krook (BGK) approximation (Bhatnagar *et al.* 1954) is adopted:

(6.2) $$\Omega (g)=-\frac{1}{\tau }\left(g-g^{eq}\right),$$

where *τ* is the relaxation time (linked to viscous effects) and *g^eq^* is the equilibrium distribution derived from the Maxwell–Boltzmann distribution.

In the LBM, the distribution function is discretised in velocity space, yielding a finite set of discrete populations *g*_*i*_ associated with discrete velocities *ξ_i_*. Transport and collisions are solved on a Cartesian lattice (voxel mesh). The present simulations employ the D3Q19 scheme, where “D3” indicates three spatial dimensions and “Q19” the number of discrete velocity directions (Qian *et al.* 1992). The macroscopic density *ρ* and momentum *ρ**u*** are obtained from moments of the discrete populations:

(6.3) $$\rho (x,t)=\sum_{i}g_{i}(x,t),\;\rho u(x,t)=\sum_{i}\xi_{i}g_{i}(x,t).$$

A very-large-eddy simulation (VLES) strategy is employed, in which the large turbulent scales are resolved while the unresolved scales are modelled through an effective relaxation time. In PowerFLOW, the sub-grid contribution is incorporated by augmenting the viscous relaxation time with a turbulent relaxation time based on a renormalisation group *k*–ϵ model (Yakhot & Orszag 1986):

(6.4) $$\tau_{\text{eff}}=\tau +C_{\mu }\frac{k^{2}/\epsilon }{{\left(1+\zeta^{2}\right)}^{1/2}},$$

where *C*_*μ*_ = 0.09, *k* and *ϵ* are the turbulent kinetic energy and dissipation rate, and *ζ* is a local parameter combining strain, vorticity and helicity contributions (Texeira 1998). The role of *ζ* is to mitigate the sub-grid viscosity in the presence of strong resolved vortical structures. Unlike Reynolds-averaged Navier-Stokes closures, which directly prescribe Reynolds stresses, the LBM–VLES approach modifies the local relaxation properties of the kinetic equation (and thus the effective eddy viscosity); the Reynolds stresses emerge from the resolved unsteady motion rather than being imposed explicitly (Chen *et al.* 2004).

Near-wall turbulence is treated using an extended turbulent wall model that accounts for pressure-gradient effects (PGE-WM) (Texeira 1998). The model rescales the wall coordinate in the generalised law-of-the-wall (Launder & Spalding 1974) through a pressure-gradient-dependent factor *A*:

(6.5) $$u^{+}=\frac{1}{\kappa }\ln\left(\frac{y^{+}}{A}\right)+B,$$

where *k* is the von Kármán constant, *B* is an additive constant, and *y*^+^ = *u*_*τ*_ *y* / *v*. The parameter *A* accounts for the slowing down and thickening of the near-wall profile under adverse pressure gradients and is defined as (Texeira 1998)

(6.6) $$\begin{array}{cc} A=1+\frac{\beta |\text{d}p/\text{d}s|}{\tau_{w}}, & u\cdot \nabla p>0, \end{array}$$

(6.7) $$\begin{array}{cc} A=1, & \;\text{otherwise,} \end{array}$$

where *τ*_*w*_ is the wall shear stress, d*p*/d*s* is the streamwise pressure gradient, ***u*** is the mean velocity vector, and *β* is a length scale of the order of the unresolved near-wall region.

### 6.2 Numerical configurations under study

**Figure 15:**
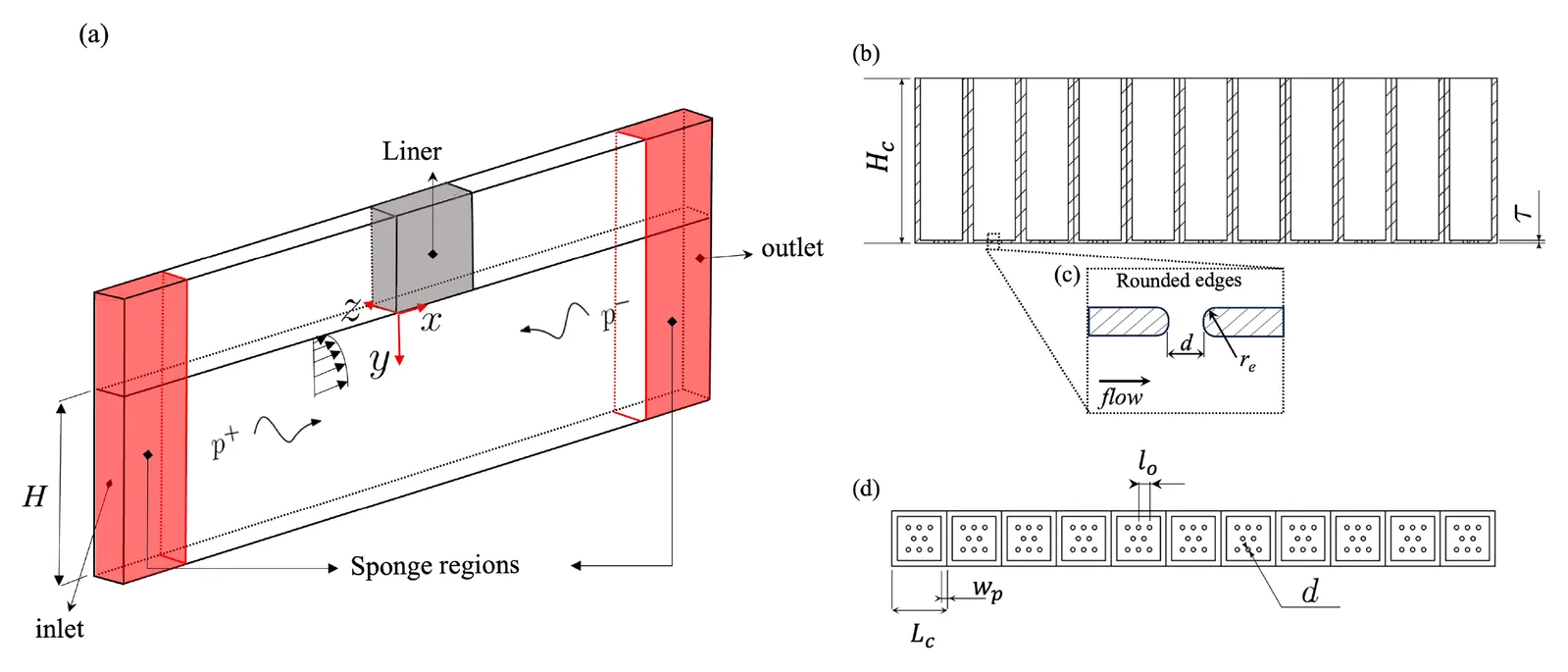
Schematic representation of (a) computational domain, (b,c) cross-sectional view of the liner and (d) wall-normal view of the liner surface.

The computational domain (Figure 15) reproduces the UFSC liner test rig (Quintino *et al.* 2025), whose properties are quite close to those of the sample used in the B2A facility. The liner is mounted on the top wall at mid-channel. Each cell has a square cross-section of side length *L*_*c*_ = 12.44 mm and depth *H*_*c*_ = 38.1 mm, and *d* = 1.17 mm is the orifice diameter. Each cavity contains eight orifices, with centre-to-centre spacing *l*_*o*_ = 2.34 mm; the partition-wall half-thickness is *w*_*p*_ = 1.27 mm and the face-sheet thickness is *τ* = 0.54 mm, yielding an overall open-area ratio of 5.5%. The duct height is *H* = 2*h* = 40 mm and its width is equal to *L*_*c*_. Another important feature of the test geometry is that the orifices have rounded edges with a radius *r*_e_ = 0.066 mm.

To match the measured upstream velocity profile, a zig-zag trip is installed on both walls at *x* = − 1.60 m (with *x* = 0 at the liner leading edge), with height 0.25 mm and length 2 mm. To minimise acoustic reflections, sponge regions are applied near the outlet (see figure 15) by increasing the viscosity up to 100 times according to an exponential law. All walls are treated as adiabatic, and periodic boundary conditions are imposed on the lateral side walls. A uniform inflow corresponding to *M*_*b*_ = 0.3 is prescribed, yielding a centreline velocity *U*_0_ ≃ 110 m/s (*M*_*b*_ = 0.32) in the lined section, while a pressure boundary condition is imposed at the outlet.

The mesh is built using a variable resolution (VR) symmetric with respect to the centre of the channel. The highest resolution, variable resolution of 7, was used to discretise the entire face sheet, the orifices, and portions of the back cavities. Each subsequent resolution level was defined by doubling the cell size of the previous level. Within the orifice, the minimum grid spacing in wall units is equal to *Δx*^+^ = *Δy*^+^ = *Δz*^+^ = 6.9, where *Δx*^+^ = *Δxu*_*τ*_/*v* and $u_{\tau }=\sqrt{\tau_{w}/\rho }=4.2$ m/s refers to the friction velocity of the smooth reference surface. Details of the mesh-convergence study are reported in Paduano *et al.* (2026).

The presented simulations follow a two-step approach. Firstly, a turbulent flow simulation is performed to obtain a statistically stationary flow field within the duct. Upon achieving convergence, an instantaneous snapshot of the flow is extracted, onto which the acoustic perturbation is then superimposed, with an excitation frequency of 1400 Hz. This seeded field serves as the initial condition for the subsequent simulation phase. During this second phase, the interaction between the acoustic field and turbulent flow is explicitly resolved, as opposed to approaches where the acoustic perturbation is linearised around the mean-flow solution (Kierkegaard *et al.* 2010).

This methodology allows for capturing the inherently nonlinear response of the liner when exposed to grazing acoustic wave at high SPL, since the interaction between the unsteady turbulence and the imposed acoustic field is directly computed rather than assumed to be linear a priori.

### 6.3 Flow and acoustics characterization

**Figure 16:**
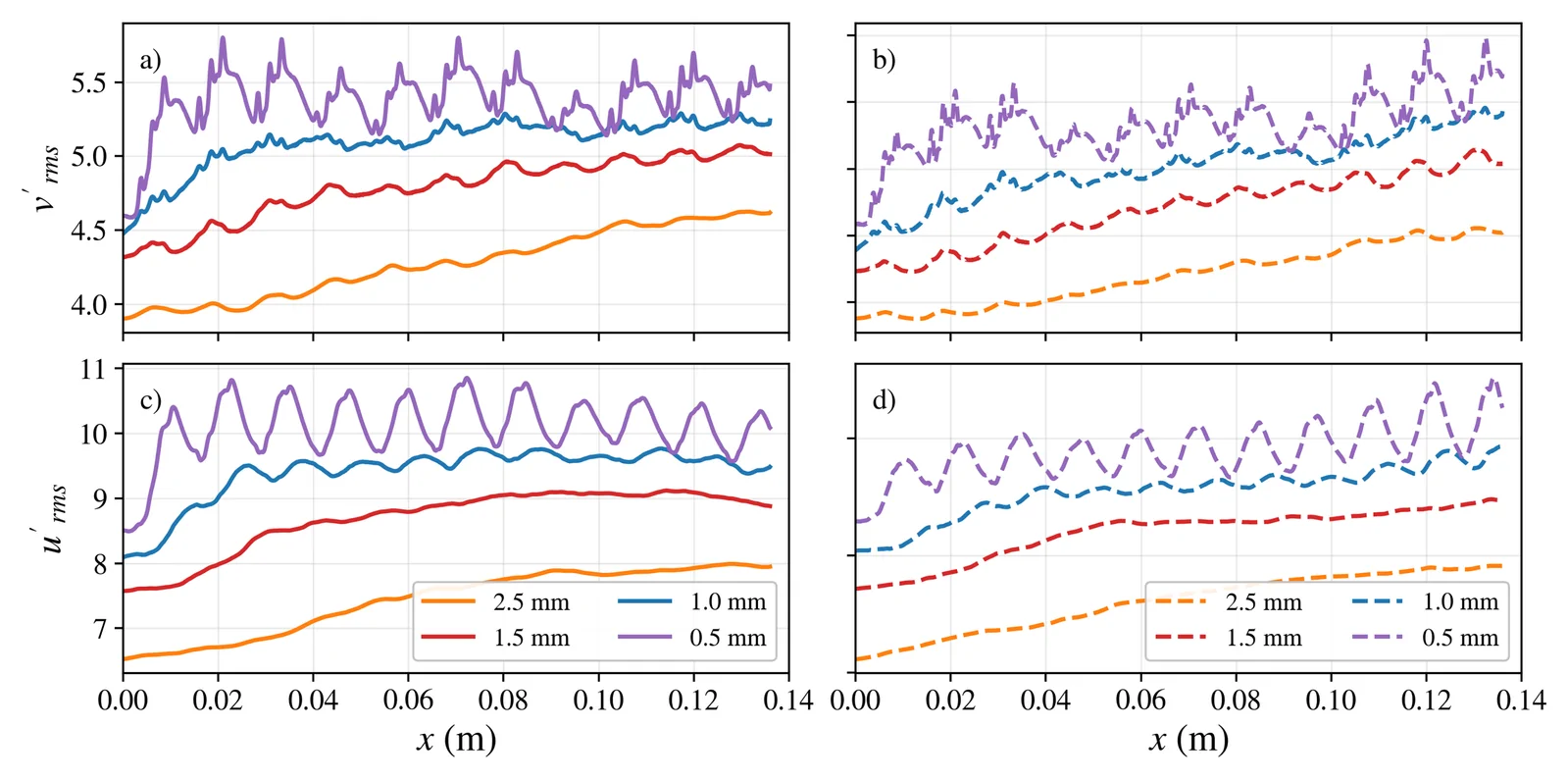
The rms of the wall-normal (a, b) and streamwise (c, d) velocity fluctuations above the liner for an acoustic excitation at SPL = 145 dB and *f* = 1400 Hz. (a, c) upstream source. (b, d) downstream source.

Figure 16 reports the streamwise evolution of the spanwise-averaged velocity fluctuations, $v_{\text{rms}\;}^{\prime }$ and $u_{\text{rms}\;}^{\prime }$, evaluated on four wall-normal planes. Results are shown for the upstream acoustic-source configuration (figure 16*a,c*) and for the downstream-source configuration (figure 16*b,d*). Across all planes and for both source locations, the distributions highlight the strong influence of the imposed SPL on the unsteady flow over the lined section.

For the upstream-source case, the wall-normal fluctuations measured closest to the wall (*y* = 0.5 mm) exhibit a marked spatial modulation that is periodic with the liner cell length, *L*_*c*_. Within each cavity, three local maxima can be distinguished; their streamwise spacing matches the orifice arrangement inside the cavity. This indicates that, in the immediate vicinity of the wall, $v_{\text{rms}\;}^{\prime }$ retains a clear imprint of the liner geometry. As the sampling plane is moved away from the wall, the amplitude of this modulation decreases and the imprint of the orifice pattern becomes progressively less distinct, consistent with a rapid wall-normal homogenization of the orifice-induced fluctuations.

A different behaviour is observed on the outermost plane (*y* = 2.5 mm), where the periodic modulation is no longer visible and the streamwise trend is instead characterised by the largest overall increase in $v_{\text{rms}\;}^{\prime }$. This change suggests a transition from a near-wall response dominated by the localised forcing associated with the perforations to a more spatially distributed fluctuation field in the outer part of the boundary layer. In this region, the streamwise growth of $v_{\text{rms}\;}^{\prime }$ is consistent with enhanced wall-normal transport associated with secondary motions developing downstream of the smooth-to-lined interface, which may increase the displacement thickness and redistribute turbulent fluctuations (Paduano *et al.* 2026).

The streamwise fluctuations $u_{\text{rms}\;}^{\prime }$ display similar near-wall modulation. Close to the liner, $u_{\text{rms}\;}^{\prime }$ varies periodically with characteristic length *L*_*c*_, reflecting the streamwise non-uniformity introduced by the cavity pattern. With increasing wall-normal distance, the periodic modulation weakens and the profiles become smoother. As for $v_{\text{rms}\;}^{\prime }$, the largest net increase in $u_{\text{rms}\;}^{\prime }$ occurs on the outermost plane (*y* = 2.5 mm), consistent with the same redistribution mechanisms discussed above. The concurrent amplification of wall-normal and streamwise velocity fluctuations suggests enhanced momentum exchange between the near-wall region and the outer flow. In particular, the increase in $v_{\text{rms}\;}^{\prime }$ is accompanied by a corresponding increase in $u_{\text{rms}\;}^{\prime }$, indicating a progressive displacement of the flow away from the wall. Such behaviour is reminiscent of turbulent boundary layers subjected to wall-normal blowing or developing over permeable surfaces, where transpiration-induced motions modify the near-wall velocity field and increase the effective displacement thickness (Jiménez 2004; Rosti *et al.* 2015; Medjnoun *et al.* 2020). In the upstream-source configuration, this growth is most pronounced over the first portion of the lined section and then tends to level off further downstream, in line with the concurrent decay of the SPL along the liner.

For the downstream-source case, the main features of $v_{\text{rms}\;}^{\prime }$ are preserved: near the wall the fluctuations remain modulated at the liner cavity length, whereas farther from the wall the modulation weakens and a net streamwise increase is observed on the outer plane. The most noticeable difference concerns $u_{\text{rms}\;}^{\prime }$. Although the overall behaviour remains similar to that of the former case, its streamwise development depends more strongly on position along the liner. At *y* = 0.5 mm the periodic pattern persists, but its amplitude increases toward the downstream end of the liner, where the SPL is highest for this source location. On planes farther from the wall (*y* = 2.5 mm), $u_{\text{rms}\;}^{\prime }$ exhibits a more monotonic streamwise growth, contrasting with the upstream-source case where the increase becomes asymptotic near the end of the lined section. This comparison indicates that the spatial distribution of acoustic forcing, set by the source location and the resulting SPL evolution, modulates both the near-wall response and the outer-layer development of the fluctuating field.

### 6.4 Data analysis with the DIR-TDIBsC

**Figure 17:**
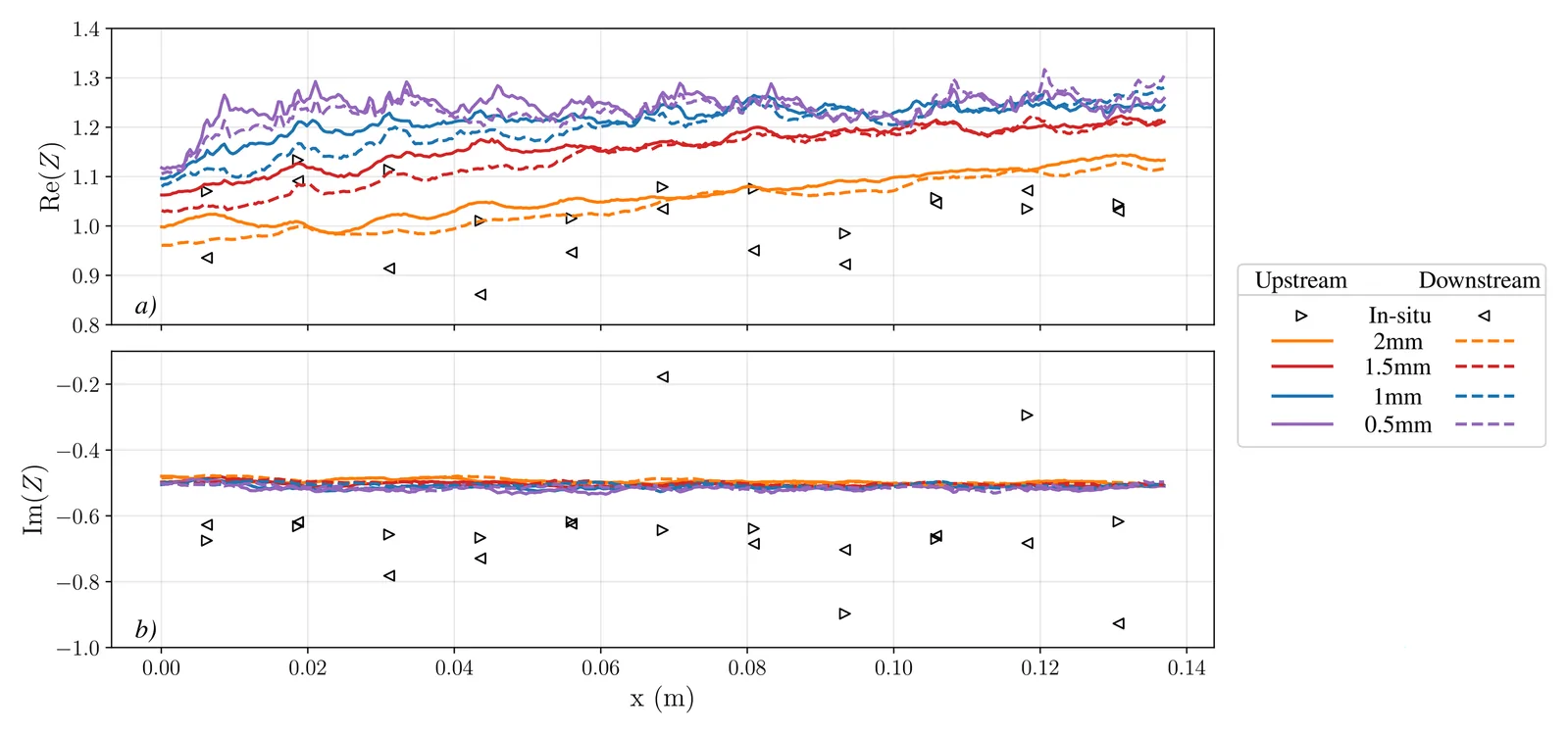
The DIR-TDIBC-based eduction using LES velocity fields. Comparison of the impedance at 1400Hz as a function of the streamwise direction, for different plane heights above the liner. a) Resistance. b): Reactance. Black symbols correspond to a direct evaluation of the admittance-averaged impedance obtained over the liner, using the in-situ method described by Paduano *et al.* (2026) for the same simulation, over each cavity.

The DIR-TDIBC approach was applied to wall-normal velocity data sampled at various plane heights above the liner, following the methodology established in Section 5.5. However, due to the limited physical duration of the simulation relative to the LDV experiments, the signals could not be segmented into smaller temporal windows for statistical analysis. The spatially varying impedance results for both upstream- and downstream-source configurations are presented in figure 17.

A primary observation is the minimal discrepancy between impedances derived from upstream and downstream excitations, regardless of the measurement plane height. Furthermore, consistent with the LDV experimental findings, the influence of the grazing flow dominates over acoustic effects. Specifically, the resistance monotonically increases in the streamwise direction, while the reactance exhibits a slight decrease.

Near the leading edge of the liner (*x* = 0), the resistance under downstream excitation is slightly lower than that of the upstream configuration. This is physically consistent: at this location, the flow-induced fluctuations have not yet fully developed over the liner’s surface, while the acoustic energy from the downstream source has already undergone significant attenuation. Conversely, for the upstream source, the acoustic contribution remains undamped at the leading edge, accounting for the observed difference.

The resistance values obtained here differ substantially from the experimental results in Section 5.5. Part of this discrepancy is attributed to differences in liner geometry between the experimental and numerical configurations. In particular, the global liner porosity is approximately 39% higher in the numerical configuration, which directly affects both the linear and nonlinear impedance characteristics. Additional discrepancies are likely rooted in the fundamental differences between the simulated and experimental flow fields.

Using the in-situ method on the LES data at the liner wall location, it is possible to extract the impedance at each mesh cell location, as shown by Paduano *et al.* (2026). The obtained impedances were averaged spatially (based on the admittance averaging) over each liner cell, and reported in figure 17. A good agreement with the spatially-varying impedances obtained using the DIR-TDIBC approach is obtained. Since the in-situ method is point-wise and based on the face-sheet and backplate pressure (Schuster 2012; Zhang & Bodony 2016b), the educed impedance is sensitive to the streamwise sampling location relative to the orifices (Avallone & Casalino 2021), and to the direction of acoustic propagation (Paduano *et al.* 2026), which is consistent with the small upstream/downstream discrepancies observed in figure 17, even though the mismatch remains small compared with that obtained with the direct eduction approach, as shown in Section 6.4.2.

These results confirm that upstream and downstream configurations do not produce divergent acoustic signatures near the liner wall. This reinforces the hypothesis that the upstream-downstream mismatch observed in other studies may stem from modelling biases in the eduction methods rather than from an inherent physical change in the impedance itself.

#### 6.4.1 Comparison of predictions with pressure microphone measurements

Similarly to what was done in Section 5.5.4, the accuracy of the educed impedances is assessed by comparing numerical predictions with acoustic pressure probes evaluated on the wall opposite to the liner. The LEEs are solved in a two-dimensional domain using a discontinuous Galerkin (DG) method to simulate the acoustic propagation in the frequency domain. The liner’s impedance is taken as that obtained via the DIR-TDIBC method (spatially averaged based on the admittance), and evaluated at each of the four sampling planes. This is done in order to quantify the sensitivity of the results to the measurement distance from the liner.

In the LEE set-up, the shear flow profile of Rienstra & Vilenski (2008, Eq. 4) is taken, with a shear parameter of 0.1. The flow profile is adjusted to match the bulk velocity of the LES.

**Figure 18:**
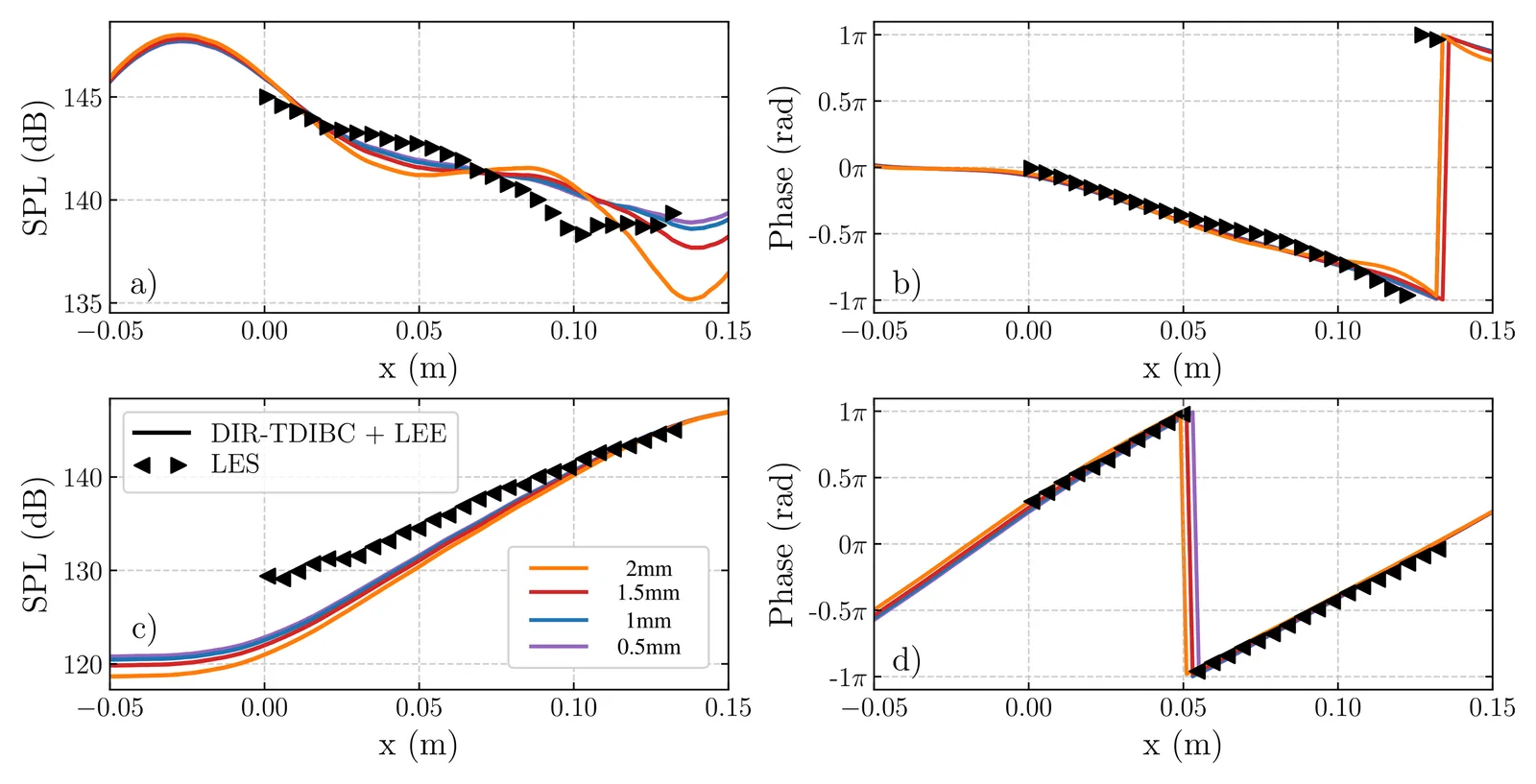
Comparison of acoustic pressure—(a, c) SPL and (b, d) phase—between high-fidelity microphone probes in the PoliTo LES simulation at Mach 0.3 (symbols) and numerical predictions from a two-dimensional LEE solver (lines), using the impedances stemming from the DIR-TDIBC approach of Section 6.4, evaluated at the four sampling planes. (a, b) Upstream-source case. (c, d) Downstream-source case. Flow goes from left to right. Liner located between *x* = 0m and *x* = 0.137m.

The results, presented in Figure 18, demonstrate correct agreement between the theoretical predictions and LES data. They also indicate that the numerical solutions obtained using the different impedances associated with each measurement distance in the DIR-TDIBC framework remain relatively close to one another.

#### 6.4.2 Direct eduction approach

To provide a comparison with the previous DIR-TDIBC results, pressure probes located on the wall opposite the liner were also analysed using a classical direct eduction approach based on the KT algorithm, in the frequency domain, similarly to what was done in Section 5.5.5. This method fits a wavenumber to the pressure measurements directly above the liner and uses the resulting relationship between the wavenumber and the impedance to retrieve *Z*, assuming a constant mean flow.

**Figure 19:**
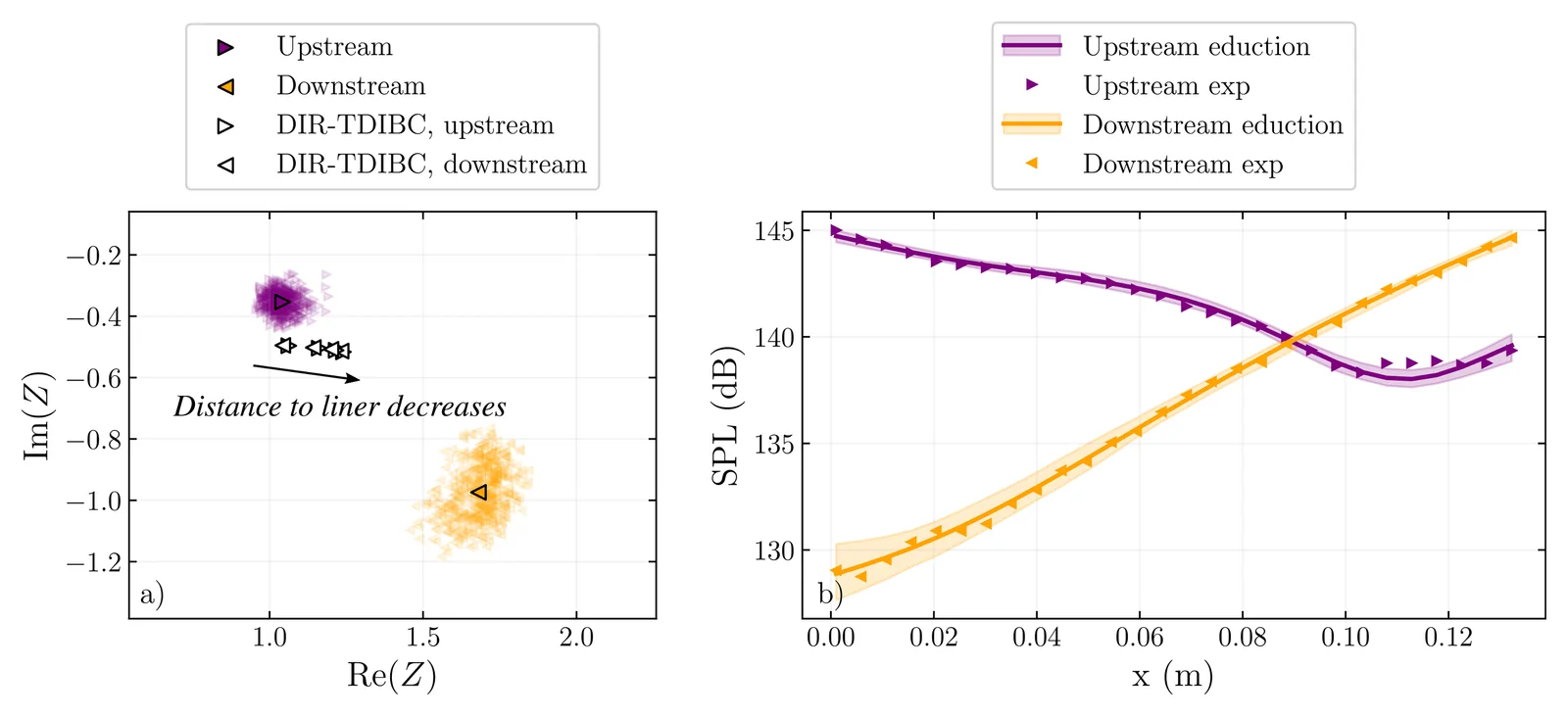
Results of the direct eduction approach at Mach 0.3 with an excitation frequency of 1400 Hz, based on pressure probes located on the wall opposite the liner. (a) Impedances in the complex plane for each sample and their mean. The symbols associated with the DIR-TDIBC method indicate impedances obtained for different plane locations ranging from 0.5 to 2 mm above the liner surface; the arrow denotes the trend observed as the sampling plane approaches the liner.. (b) Corresponding comparison of measured and predicted SPL above the liner, where the values have been shifted to start at 145dB. The shaded envelope denotes the minimum and maximum limits across samples. Flow goes from left to right. Liner located between *x* = 0m and *x* = 0.137m.

The resulting impedances and SPL fits are shown in figure 19. Once again, despite good agreement between the measured and modelled pressures, the retrieved impedances exhibit significant differences between the upstream and downstream configurations. The impedance obtained in the upstream configuration is 1.04− 0.36j, while the downstream configuration yields 1.68− 0.98j.

Similarly to the experimental case of Section 5, the DIR-TDIBC impedances, evaluated using velocity probes located at four planes ranging from 0.5 mm to 2 mm above the liner, remain very consistent with each other for a given wall-normal position. As the measurement plane approaches the liner, these estimates tend to converge towards values lying close to the line connecting the upstream and downstream impedances obtained with the KT algorithm.

## 7 Discussion

This section regroups different elements of discussions relating to the merits and drawbacks of the method, as well as to potential research perspective seeds for the future.

### 7.1 Spatial locality and impedance heterogeneity

The proposed method is inherently spatially local, providing approximate impedance values directly at each measurement location. This locality constitutes a major advantage when investigating spatial variations of liner impedance, which are difficult to access with classical eduction techniques that rely on spatially homogenised boundary conditions.

### 7.2 Choice of measurement distance and homogenization issues

One potential drawback of the method concerns the choice of the distance from the liner at which the velocity is extracted. If the measurement is performed too far from the liner, the velocity signal may lose fidelity and no longer accurately represent the conditions at the liner surface. Conversely, if the measurement is taken too close to the liner, strong spatial discontinuities arise due to the local orifice impedance. In this case, the method cannot distinguish whether the measured signal corresponds to a region above a perforation or a solid wall, which may bias the educed impedance. The framework could in principle be applied above a different surface or even a rigid wall and still produce an impedance estimate. This behaviour reflects the fact that the method does not directly measure impedance, but rather evaluates the impedance that is consistent with the assumed nonlinear model and the local velocity field.

Establishing guidelines for an optimal measurement distance therefore remains an open research question and is expected to depend on the liner geometry, the SPL, and the Mach or Reynolds number. In particular, the method is expected to be more robust at higher Mach numbers, where increased mixing near the liner perforations tends to reduce the homogenization distance. This observation is noteworthy, as this operating regime is precisely where classical impedance eduction approaches often encounter difficulties. Appendix A establishes the basis for this future study.

An alternative approach would consist of developing a coherence-based filtering strategy, in which velocity signals exhibiting low coherence with the acoustic excitation would be interpreted as originating from wall regions (admittance taken as 0), whereas signals with high coherence would be considered suitable inputs for the time-domain method developed in this work.

### 7.3 Frequency range and excitation dependence

While the method can be applied at the excitation frequency used in the experiment, it can also target other frequencies or excitation types, such as swept-sine or multi-tone signals. This extension is valid only under the assumption that the acoustic excitation does not significantly alter the flow dynamics in the measurement region, an assumption that remains debatable in strongly coupled flow**–**acoustic regimes.

Nevertheless, the time-resolved nature of LDV measurements allows the proposed method to access a broad spectral range with high frequency resolution. A significant advantage of the high-frequency capability of the method is that distinguishing between plane-wave and higher-order modal content in the duct is no longer required. For instance, in the B2A facility, multiple modes are cut on above 3 000 Hz, yet this does not hinder the applicability of the method in this frequency range.

The only additional requirement to fully exploit this capability would be to perform LDV measurements in the transverse direction as well, in order to reconstruct a two-dimensional impedance map rather than a single measurement line. This situation is already available in the high-fidelity simulations considered in the present work, although only plane-wave excitations have been analysed so far.

### 7.4 Experimental limitations of LDV-based measurements

In practice, LDV measurements performed close to the liner may suffer from reduced acquisition rates, sometimes severely, due to limitations associated with flow seeding. For liners with micro-perforations or wire meshes, clogging by seeding particles represents an additional limitation.

In such cases, the method is feasible only if a bias flow is introduced to prevent clogging of the perforations. We note that liner bias flows are inherently interesting due to their widespread use in combustion chambers and related applications for transpiration cooling.

### 7.5 Model dependence and applicability to complex liners

The proposed approach relies on the availability of an adequate liner impedance model, including both its linear and nonlinear frequency-domain response. In other words, the method presumes prior knowledge of the liner behaviour and primarily serves to identify the realised impedance under given operating conditions.

As the method has so far been applied to a limited set of samples and configurations, the observed agreement with classical approaches should be further confirmed by additional studies. At present, the method appears best suited for SDoF liners, for which the time-domain nonlinear behaviour is relatively well characterised.

For more complex liners, such as double-degree-of-freedom (DDoF) configurations consisting of stacked SDoF elements, it would be necessary to explicitly account for the nonlinear behaviour of the second perforated plate in order to accurately assess the liner impedance, for instance using the work of Schwoebel *et al.* (2026). This constitutes an important direction for future work.

## 8 Conclusions

This work introduced a direct, time-domain approach for the approximate eduction of acoustic liner impedance in the presence of turbulent grazing flows. By combining velocity measurements taken in the immediate vicinity of the liner with the DIR-TDIBC framework, the proposed method bypasses the need for an acoustic wave-propagation model and instead relies on local flow information and a time-domain impedance formulation. This represents a conceptual departure from classical eduction strategies, in which the impedance is inferred indirectly through its effect on global acoustic fields at a given frequency. Beyond its methodological contribution, the approach was specifically developed to investigate two long-standing open questions in liner acoustics: the physical origin of the flow effect on impedance and the upstream–downstream impedance mismatch reported in conventional eduction methods.

The DIR-TDIBC method introduced in Section 3 operates as follows. The reflection coefficient of the liner is expressed as a finite-length impulse response array, obtained via the inverse Fourier transform of the frequency-domain reflection coefficient. This impulse response is updated at each time step to account for the time-varying velocity, allowing nonlinear effects to be captured dynamically. The reflected wave is then reconstructed through a time-local convolution between the incident acoustic wave and this evolving impulse response kernel. In the presence of turbulent grazing flow, the total wall-normal velocity—comprising mean, hydrodynamic, and acoustic contributions—is used directly to drive the nonlinear response, under the assumption that turbulent fluctuations contribute to nonlinearity in a manner analogous to high-amplitude acoustic excitation.

The approach was applied to both experimental LDV measurements acquired in the B2A aeroacoustic facility at ONERA and high-fidelity LES data obtained at PoliTo. In both contexts, the method yielded impedance estimates that are consistent with established semi-empirical models and frequency-based estimates, while providing additional insight into the spatial variability of the liner response under grazing-flow conditions. In particular, the time-domain formulation naturally captures amplitude-dependent nonlinear effects associated with large particle velocities at the liner surface, and can be applied at frequencies far removed from the main loudspeaker excitation frequencies.

A central motivation of this study was to determine whether the upstream**–**downstream mismatch in educed impedance, widely reported in the literature, stems from limitations of wave-propagation modelling or from a genuine physical asymmetry in liner**–**flow interactions. The present results show that, once wave-propagation modelling is entirely removed from the eduction process, the differences between upstream and downstream excitation become minimal. The small residual discrepancies can be attributed to variations in turbulence intensity and in the acoustic SPL along the liner. In the configurations investigated here, the liner is found to increase the turbulence level in the streamwise direction, which in turn leads to an increase in resistance and a reduction in reactance. This effect is observed even though the dominant frequencies of the turbulence lie outside the acoustic excitation band. In addition, as the acoustic wave propagates over the liner and progressively loses energy, the effective resistance may either increase or decrease in the streamwise direction, depending on the location of the primary acoustic source. The impedances obtained via DIR-TDIBC for upstream and downstream source locations showed far less variations than the impedances obtained via the benchmark direct eduction approach. These results strongly suggest that the upstream–downstream impedance mismatch reported in the literature is not an intrinsic property of the liner, but arises predominantly from the wave-propagation assumptions embedded in the eduction process.

The experimental and numerical datasets highlighted the role of turbulence-induced velocity fluctuations in modulating the nonlinear contribution to the impedance. This constitutes the main physical finding of the present study, and supports recent suggestions that turbulent grazing flows may influence liner behaviour through mechanisms analogous to high-SPL nonlinearities, even in the absence of strong tonal excitation. From this perspective, the impedance should be viewed not as a purely material property, but as an emergent quantity resulting from the coupled dynamics of acoustics, flow, and liner micro-geometry.

The present results suggest that many existing semi-empirical models for flow effects are fundamentally facility-dependent, notably due to the yet unreported Reynolds effect onto the wall normal velocity fluctuation above the liner, an important line of future work. Furthermore, we argue that traditional corrections aimed at capturing the flow effects may have inadvertently been characterizing the nonlinear impact of those wall-normal velocity perturbations. These perturbations appear to modify the impedance in a manner physically identical to high-amplitude acoustic-driven particle velocity.

The proposed methodology offers a complementary perspective to conventional impedance eduction techniques and provides a valuable and fast diagnostic tool for disentangling propagation effects from local liner physics. Future work will focus on extending the approach to more complex liner geometries, and exploring the role of turbulence statistics in shaping the effective acoustic response. More broadly, the present results advocate for a shift towards locally resolved, time-domain analyses when addressing liner behaviour in realistic aeroacoustic environments.

### Supplementary Material

Appendix

## Figures and Tables

**Table 1:** Main characteristics of the experimental and numerical configurations analysed in this study.

Parameter	Experiment, Sec. 5	Numerical simulation, Sec. 6
Liner name	ONERA sample	PoliTo sample
Configuration origin	ONERA B2A facility	UFSC liner test rig model
Duct cross-section	50 × 50 mm square	40 mm height, periodic width
Liner type	SDoF	SDoF
Liner length *L*	150 mm	137 mm
Cavity depth *H_c_*	38.1 mm	38.1 mm
Cavity side length *D*	9.91 mm	9.90 mm
Cell pitch *L_c_*	12.44 mm	12.44 mm
Cavity wall half thickness *w_p_*	1.27 mm	1.27 mm
Facesheet thickness *τ*	0.635 mm	0.54 mm
Orifice diameter *d*	0.991 mm	1.17 mm
Number of orifices per cavity	8	8
Orifice spacing *l*_0_	3.31 mm	2.34 mm
Orifice shape	Chamfered edges	Rounded edges
Global porosity	3.95%	5.5%
Local porosity	6.5%	8.78%
Flow Mach number	0.1, 0.3	≈ 0.3
Liner Resonance Frequency	≈ 1450*H_z_*	≈ 1400*H_z_*
Acoustic excitation frequency	1500 H*z*	1400 H*z*
Acoustic excitation level	115–145 dB	145 dB
Measurement zones	*y* = 1–2.5 mm lines	*y =* 0.5–2.5 mm planes
Measured quantity	LDV velocity field	Resolved velocity field
Reference impedance availability	Indirect (eduction)	Direct (in-situ) and indirect (eduction)

**Table 2:** Experimental configurations studied by LDV in the B2A facility.

Experimental configuration	Mach	SPL (incident) (dB)	Loudspeaker position
1	0.1	115	Upstream
2	0.1	130	Upstream
3	0.1	145	Upstream
4	0.3	145	Upstream
5	0.3	145	Downstream

## Data Availability

Raw LDV data were generated at ONERA, while raw LBM-VLES data were generated at PoliTo. Derived data supporting the findings of this study are available upon reasonable request from the corresponding author RR.
