# Supplementary material for: Direct Time-Domain Eduction of Acoustic Liner Impedance in Turbulent Grazing Flows
Source: J Fluid Mech. Author manuscript; Available in PMC 2026 Sep 11. (PMC7619478; doi:10.1017/jfm.2026.11969)
Supplement: Supplementary file 1 [file EMS217255-supplement-Appendix.pdf]

Appendix A. Considerations of homogenization of liner impedance

A.1. *Purely acoustic homogenization in a grazing-incidence duct*

Even in the absence of turbulent flow, directly recovering the liner impedance from the acoustic velocity measured at a finite distance above the liner remains questionable. To assess the order of magnitude of the error introduced by this approach in the present work, we developed a simplified two-dimensional model representative of the B2A bench, in which the liner impedance is spatially discontinuous.

In this model, each perforation is assigned its own impedance. The impedance patches are 1 mm long and spaced by 3 mm, over a total length of 15 cm representative of the tested liner. This configuration captures the main geometrical features of the experimental liner while remaining computationally tractable.

The numerical simulations are performed using the LEEs. To recover the impedance, pressure and velocity are extracted along a line parallel to the liner surface at a prescribed height above it. The admittance is then spatially averaged along the liner, and the inverse of this mean admittance is taken as the homogenised impedance. This procedure is repeated for several distances from the liner.

Figure 20 shows the vertical component of the acoustic velocity field, highlighting the strong spatial discontinuities induced by the very low impedance of the perforations ($Z_p = 0.2$). The corresponding spatially averaged normalised impedance is predicted to be $Z_{\text{tot}} = 0.8$, which is of the same order of magnitude as the values obtained experimentally at Mach 0.1 in the present study.

Figure 21 presents the homogenised impedance as a function of the distance from the liner. From these results, we conclude that the LDV-based approach is expected to incur an impedance error of the order of

$$Z_{\text{err}} \approx (0.025 + \text{j}\,0.03)$$

per millimetre above the liner.

A.2. *Acoustic-driven vortex shedding and homogenization*

Following the review of Mahesh (2013, Eq. 4), we anticipate that the penetration depth of a steady jet issuing from a liner perforation scales as

$$\frac{y}{L} \propto \left(\frac{x}{L}\right)^{-1/3}, \qquad \text{(A 1}a\text{)}$$

with

$$L = (\pi/4)^{1/2}\, r d, \quad r = U_j/U_\infty, \qquad \text{(A 1}b\text{)}$$

with U_j the jet velocity and U_∞ the mean flow velocity.

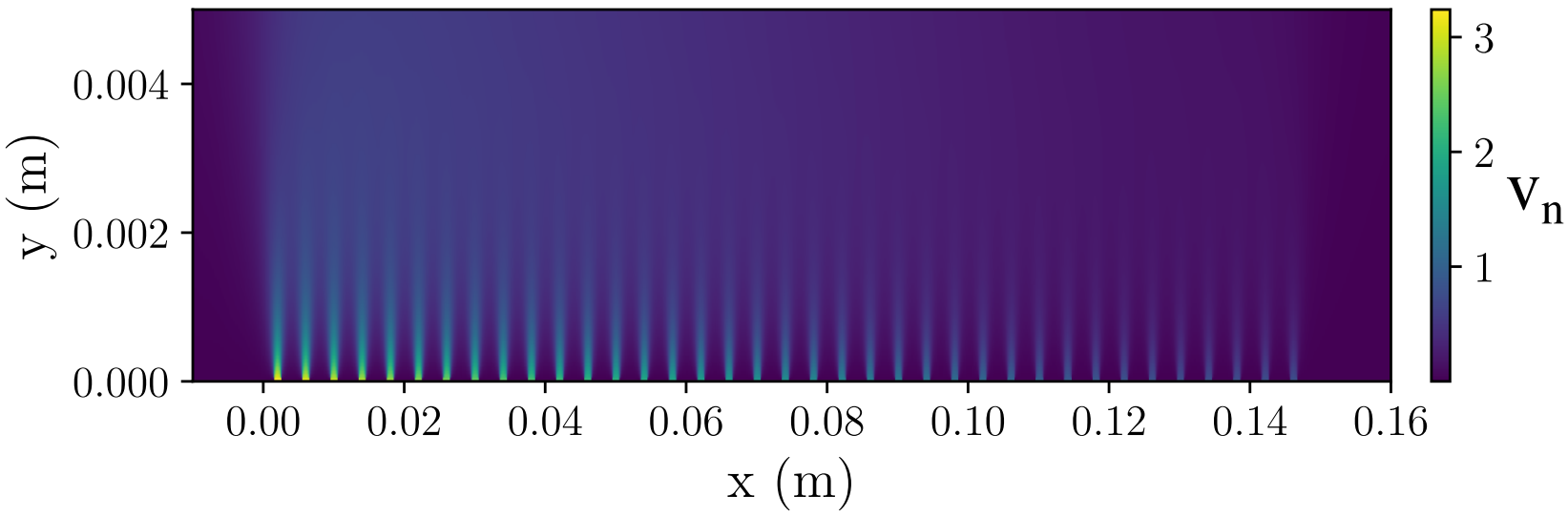

Figure 20. Magnitude of the vertical component of the velocity above a liner made of discontinuous patches of impedance, at Mach 0 and for an incident SPL of 145dB, using the LEEs and a discontinuous Galerkin, frequency-domain harmonic solver.

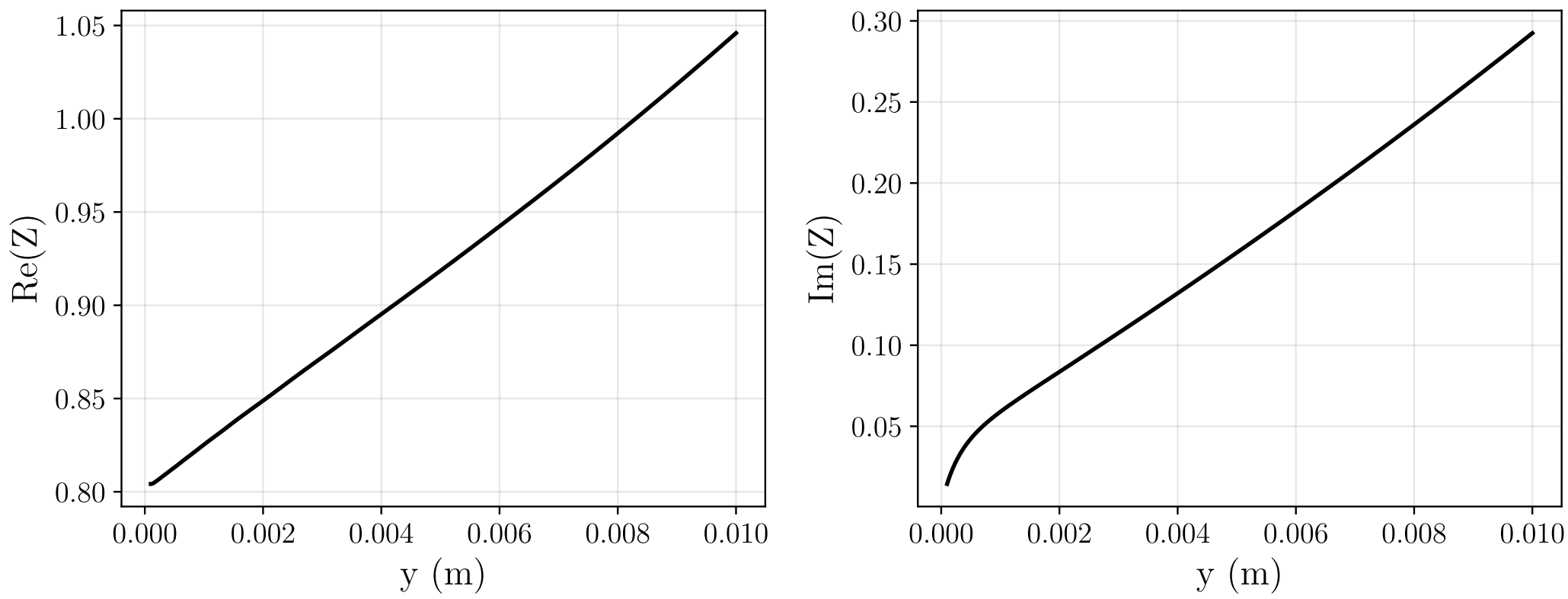

Figure 21. Homogenised impedance as a function of the distance to the liner, obtained at Mach 0, for the data extracted from the simulation depicted in figure 20.

We assume that a similar scaling holds for a pulsed jet, where the jet velocity is replaced by the rms velocity within the perforation. For a given sound pressure level (SPL) and a liner of porosity ϕ and real impedance Z_{tot}, the jet velocity U_j in the perforation can be estimated as

$$p_{\text{rms}} = \frac{p_0}{\sqrt{2}} \cdot 10^{\frac{SPL}{20}}, \tag{A 2a}$$

$$U_j = \frac{1}{\rho_f c_f} \frac{p_{\text{rms}}}{\phi |Z_{\text{tot}}|}. \tag{A 2b}$$

Considering an SPL range from 100 dB to 145 dB, a porosity between 5% and 15%, and an impedance between 0.1 and 2, the jet velocity U_j ranges from ≈ 0 m/s to ≈ 120 m/s.

From Eq. A 1a, it can be seen that the expression diverges as $x \longrightarrow \infty$. To obtain practical scaling laws relating the SPL, the Mach number, and the penetration depth of a jet-like motion at a liner orifice, we arbitrarily fix the jet penetration height H_j as the value obtained at $x_{\text{lim}} = 10d$. Given a known shear flow profile, one can then compute, for a given x_{lim} and U_j, the corresponding value of H_j using Eq. A 1a. However, at this height H_j, the maximum flow velocity is likely to be lower than the characteristic flow velocity typically used in the evaluation of Eq. A 1a. It is therefore necessary to update the velocity and repeat the calculation until convergence is achieved. The results of this convergence analysis, performed for multiple values of U_j and M_b, are presented in figure 22. The obtained scaling law reads

$$\frac{H_j}{d} \propto U_j^{0.4} M_b^{-0.5}. \tag{A 3}$$

The lower the jet penetration height H_j, the closer can LDV measurements can be taken to the liner while still yielding a homogenised impedance representation. Referring back to the results of Section 5.5.4, the increase in SPL from 130 dB to 145 dB corresponds approximately to a factor of 60 of increase in the particle velocity within the liner perforations, not factoring in the change of impedance. To reduce oscillations in the spatial

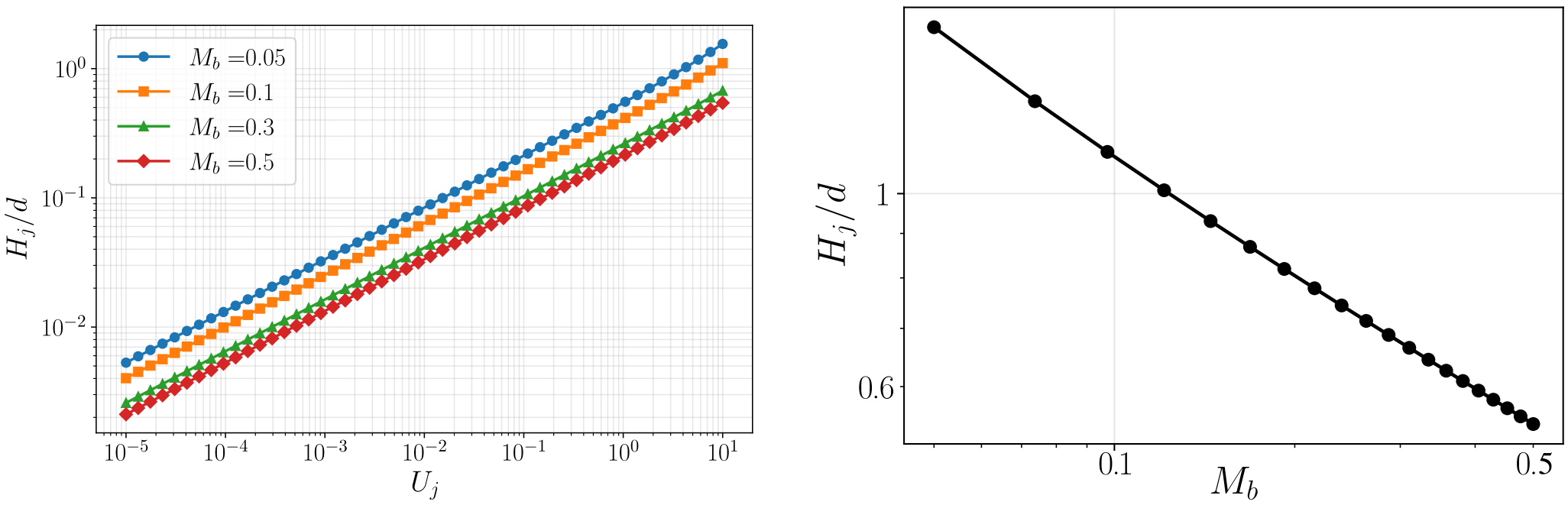

Figure 22. Dependence of the jet height with respect to jet speed and Mach number for a given shear flow parameter (δ = 0.05 in the flow profile of Rienstra & Vilenski (2008, Eq. 4)). (a) Dependence of the jet speed U_j on the jet height H_j for different bulk Mach numbers. (b) Dependence of the jet height H_j w.r.t. the bulk Mach number for U_j = 5m/s.

impedance, attributed to vortex-shedding effects (and thus to the jet-in-cross-flow mechanism studied here), the measurement height was increased by a factor of 2.5. However, based on Eq. A 3, this factor should have been closer to $60^{0.4} \approx 5$. Given that the minimum achievable distance to the liner at 130 dB is unknown, it is possible that this factor of 5 is closer to the ”true” value. In any case, it remains of the same order as that obtained experimentally, noting that measurements could not be performed closer than 1 mm from the liner.

From figure 22 and Eq. A 3, it can be observed that, at a fixed SPL of 145 dB, increasing the Mach number from 0.1 to 0.3 reduces the jet penetration height H_j by a factor of approximately $(1/3)^{-0.5} \approx 1.7$, assuming a similar flow profile shape. This implies that the results obtained at Mach 0.3 are, in terms of penetration height, roughly equivalent to those that would have been obtained at a distance of 1.7 mm above the liner at Mach 0.1.
